# Colorectal-vaginal fistula after rectal cancer resection: international comparative cohort study of characteristics and treatment

**DOI:** 10.1093/bjs/znaf189

**Published:** 2025-11-18

**Authors:** Mila L van Lieshout, Jobbe M G Lemmens, Nynke G Greijdanus, Kiedo Wienholts, Sander Ubels, Kevin Talboom, Gerjon Hannink, Albert Wolthuis, F Borja de Lacy, Jérémie H Lefevre, Michael Solomon, Matteo Frasson, Nicolas Rotholtz, Quentin Denost, Rodrigo O Perez, Tsuyoshi Konishi, Yves Panis, Martin Rutegård, Roel Hompes, Frans van Workum, Pieter J Tanis, Johannes H W de Wilt, TENTACLE–Rectum Collaborative Group, Andreas J A Bremers, Floris T Ferenschild, Stefanie de Vriendt, André D’Hoore, Gabriele Bislenghi, Jordi Farguell, Antonio M Lacy, Paula González Atienza, Charlotte S van Kessel, Yann Parc, Thibault Voron, Maxime K Collard, Jorge Sancho Muriel, Hannia Cholewa, Laura A Mattioni, Alice Frontali, Sebastiaan W Polle, Fatih Polat, Ndidi J Obihara, Bruna B Vailati, Miranda Kusters, Jurriaan B Tuynmann, Sanne J A Hazen, Alexander A J Grüter, Takahiro Amano, Hajime Fujiwara, Mario Salomon, Hernán Ruiz, Ricardo Gonzalez, Diego Estefanía, Nicolas Avellaneda, Augusto Carrie, Mateo Santillan, Diana A Pantoja Pachajoa, Matias Parodi, Manuel Gielis, Alf-Dorian Binder, Thomas Gürtler, Peter Riedl, Sarit Badiani, Christophe Berney, Matthew Morgan, Paul Hollington, Nigel da Silva, Gavin Nair, Yiu M Ho, Michael Lamparelli, Raj Kapadia, 19 Hidde M Kroon, Nagendra N Dudi-Venkata, Jianliang Liu, Tarik Sammour, Nicolas Flamey, Paul Pattyn, Ahmed Chaoui, Louis Vansteenbrugge, Nathalie E J van den Broek, Patrick Vanclooster, Charles de Gheldere, Pieter Pletinckx, Barbara Defoort, Maxime Dewulf, Mihail Slavchev, Nikolay Belev, Boyko Atanasov, Panche Krastev, Manol Sokolov, Svilen Maslyankov, Petar Gribnev, Vasil Pavlov, Tsvetomir Ivanov, Martin Karamanliev, Emil Filipov, Pencho Tonchev, Felix Aigner, Martin Mitteregger, Caterina Allmer, Gerald Seitinger, Nicola Colucci, Nicolas Buchs, Frédéric Ris, Christian Toso, Eleftherios Gialamas, Aurélie Vuagniaux, Roland Chautems, Marc-Olivier Sauvain, Silvio Daester, Markus von Flüe, Marc-Olivier Guenin, Stephanie Taha-Mehlitz, Gabriel F Hess, Lubomír Martínek, Matej Skrovina, Maria Machackova, Vladimir Benčurik, Deniz Uluk, Johann Pratschke, Luca S Dittrich, Safak Guel-Klein, Daniel Perez, Julia-Kristin Grass, Nathaniel Melling, Simone Mueller, Lene H Iversen, Jacob D Eriksen, Gunnar Baatrup, Issam Al-Najami, Thomas Bjørsum-Meyer, Jüri Teras, Roland M Teras, Fatma A Monib, Nagm Eldin Abu Elnga Ahmed, Eithar Alkady, Ahmed K Ali, Gehan Abd Elatti Khedr, Ahmed Samir Abdelaal, Fouad M Bassyouni Ashoush, Moataz Ewedah, Eslam M Elshennawy, Mohamed Hussein, Daniel Fernández-Martínez, Luis J García-Flórez, María Fernández-Hevia, Aida Suárez-Sánchez, Izaskun del Hoyo Aretxabala, Iria Losada Docampo, Jesús Gómez Zabala, Patricia Tejedor, Javier T Morales Bernaldo de Quirós, Ignacio Bodega Quiroga, Antonio Navarro-Sánchez, Iván Soto Darias, Cristina López Fernández, Cristina de La Cruz Cuadrado, Luis Sánchez-Guillén, Francisco López-Rodríguez-Arias, Álvaro Soler-Silva, Antonio Arroyo, Juan C Bernal-Sprekelsen, Segundo Á Gómez-Abril, Paula Gonzálvez, María T Torres, Teresa Rubio Sánchez, Francisco Blanco Antona, Juan E Sánchez Lara, José A Alcázar Montero, Fernando Mendoza-Moreno, Manuel Díez-Alonso, Belén Matías-García, Ana Quiroga-Valcárcel, Enrique Colás-Ruiz, Marta M Tasende-Presedo, Ignacio Fernández-Hurtado, José A Cifuentes-Ródenas, Marta Castro Suárez, Manuel Losada, Miguel Hernández, Alfredo Alonso, Beatriz Diéguez, Daniel Serralta, Rita E Medina Quintana, Jose M Gil Lopez, Francisca Lima Pinto, Elena Nieto-Moreno, Alba Correa Bonito, Carlos Cerdán Santacruz, Elena Bermejo Marcos, Javier García Septiem, Aránzazu Calero-Lillo, Javier Alanez-Saavedra, Salvador Muñoz-Collado, Manuel López-Lara, María Labalde Martínez, Eduardo Ferrero Herrero, Francisco Javier García Borda, Óscar García Villar, Jorge Escartín, Juan L Blas, Rocío Ferrer, Jorge García Egea, Antonio Rodríguez-Infante, Germán Mínguez-Ruiz, Guillermo Carreño-Villarreal, Gerardo Pire-Abaitua, Jana Dziakova, Carlos Sáez-Cazallas Rodríguez, María J Pizarro Aranda, José M Muguerza Huguet, Nerea Borda-Arrizabalaga, José M Enriquez-Navascués, Garazi Elorza Echaniz, Yolanda Saralegui Ansorena, Mercedes Estaire-Gómez, Carlos Martínez-Pinedo, Alejandro Barbero-Valenzuela, Pablo Ruíz-García, Miquel Kraft, María J Gómez-Jurado, Gianluca Pellino, Eloy Espín-Basany, Eddy Cotte, Nathalie Panel, Claire-Angéline Goutard, Nicola deÁngelis, Lelde Lauka, Shafaque Shaikh, Laura Osborne, George Ramsay, Vladimir-Ion Nichita, Santosh Bhandari, Panchali Sarmah, Rob M Bethune, Heather C M Pringle, Lisa Massey, George E Fowler, Hytham K S Hamid, Belinda D de Simone, James Kynaston, Nicholas Bradley, Roxane M Stienstra, Shashank Gurjar, Tanmoy Mukherjee, Ashfaq Chandio, Safia Ahmed, Baljit Singh, Francois Runau, Sanjay Chaudhri, Oliver Siaw, Janahan Sarveswaran, Victor Miu, Daniel Ashmore, Haitham Darwich, Deepak Singh-Ranger, Nirbhaibir Singh, Mohamed Shaban, Fahed Gareb, Thalia Petropolou, Adreas Polydorou, Mit Dattani, Asma Afzal, Akshay Bavikatte, Boby Sebastian, Nicholas Ward, Amitabh Mishra, Dimitrios Manatakis, Christos Agalianos,Nikolaos Tasis, Maria-Ioanna Antonopoulou, Ioannis Karavokyros, Alexandros Charalabopoulos, Dimitrios Schizas, Efstratia Baili, Athanasios Syllaios, Lysandros Karydakis, Michail Vailas, Dimitrios Balalis, Dimitrios Korkolis, Aris Plastiras, Aliki Rompou, Sofia Xenaki, Evangelos Xynos, Emmanuel Chrysos, Maria Venianaki, Grigorios Christodoulidis, Konstantinos Perivoliotis, George Tzovaras, Ioannis Baloyiannis, Man-Fung Ho, Simon Siu-man Ng, Tony Wing-chung Mak, Kaori Futaba, Goran Šantak, Damir Šimleša, Jurica Ćosić, Goran Zukanović, Michael E Kelly, John O Larkin, Paul H McCormick, Brian J Mehigan, Tara M Connelly, Peter Neary, Jessica Ryan, Peter McCullough, Maytham A Al-Juaifari, Hayder Hammoodi, Ali Hashim Abbood, Marcello Calabrò, Andrea Muratore, Antonio La Terra, Francesca Farnesi, Carlo V Feo, Nicolò Fabbri, Antonio Pesce, Marta Fazzin, Francesco Roscio, Federico Clerici, Andrea Lucchi, Laura Vittori, Laura Agostinelli, Maria Cristina Ripoli, Daniele Sambucci, Andrea Porta, Giovanni Sinibaldi, Giacomo Crescentini, Antonella larcinese, Emanuele Picone, Roberto Persiani, Alberto Biondi, Roberto Pezzuto, Laura Lorenzon, Gianluca Rizzo, Claudio Coco, Luca D’Agostino, Antonino Spinelli, Matteo M Sacchi, Michele Carvello, Caterina Foppa, Antonino Spinelli, Matteo M Sacchi, Michele Carvello, Caterina Foppa, Annalisa Maroli, Gian M Palini, Gianluca Garulli, Nicola Zanini, Paolo Delrio, Daniela Rega, Fabio Carbone, Alessia Aversano, Giovanni Pirozzolo, Alfonso Recordare, Lucrezia D'Alimonte, Chiara Vignotto, Carlo Corbellini, Gianluca M Sampietro, Leonardo Lorusso, Carlo A Manzo, Federico Ghignone, Giampaolo Ugolini, Isacco Montroni, Franceso Pasini, Francesco Pasini, Michele Ballabio, Pietro Bisagni, Francesca T Armao, Marco Longhi, Omar Ghazouani, Raffaele Galleano, Nicolò Tamini, Massimo Oldani, Luca Nespoli, Arcangelo Picciariello, Donato F Altomare, Giovanni Tomasicchio, Giuliano Lantone, Fausto Catena, Mario Giuffrida, Alfredo Annicchiarico, Gennaro Perrone, Ugo Grossi, Giulio A Santoro, Giacomo Zanus, Alessandro Iacomino, Simone Novello, Nicola Passuello, Martino Zucchella, Lucia Puca, Maurizio deGiuli, Rossella Reddavid, Stefano Scabini, Alessandra Aprile, Domenico Soriero, Emanuela Fioravanti, Matteo Rottoli, Angela Romano, Marta Tanzanu, Angela Belvedere, Nicolò M Mariani, Andrea P Ceretti, Enrico Opocher, Gaetano Gallo, Giuseppe Sammarco, Gilda de Paola, Salvatore Pucciarelli, Francesco Marchegiani, Gaya Spolverato, Gianluca Buzzi, Salomone Di Saverio, Paola Meroni, Cristiano Parise, Elisa I Bottazzoli, Pierfrancesco Lapolla, Gioia Brachini, Bruno Cirillo, Andrea Mingoli, Giuseppe Sica, Leandro Siragusa, Vittoria Bellato, Daniele Cerbo, Carlo A de Pasqual, Giovanni de Manzoni, Maria A di Cosmo, Bourhan M H Alrayes, Mahmoud W M Qandeel, Mohammad Bani Hani, Alexander Rabadi, Mohammad S el Muhtaseb, Basel Abdeen, Fahed Karmi, Justas Žilinskas, Tadas Latkauskas, Algimantas Tamelis, Ingrida Pikūnienė, Vygintas Šlenfuktas, Tomas Poskus, Marius Kryzauskas, Matas Jakubauskas, Saulius Mikalauskas, Lina Jakubauskiene, Soha Y Hassan, Amani Altrabulsi, Eman Abdulwahed, Reem Ghmagh, Abdulqudus Deeknah, Entisar Alshareea, Muhammed Elhadi, Saleh Abujamra, Ahmed A Msherghi, Osama W E Tababa, Mohammed A Majbar, Amine Souadka, Amine Benkabbou, Raouf Mohsine, Sabrillah Echiguer, Paulina Moctezuma-Velázquez, Noel Salgado-Nesme, Omar Vergara-Fernández, Juan C Sainz-Hernández, Francisco E Alvarez-Bautista, Andee D Zakaria, Zaidi Zakaria, Michael P K Wong, Razif Ismail, Aini F Ibrahim, Nik A N Abdullah, Rokayah Julaihi, Sameer Bhat, Greg O'Grady, Ian Bissett, Bas Lamme, Gijsbert D Musters, Anne M Dinaux, Brechtje A Grotenhuis, Ernst J Steller Arend G J Aalbers, Marjolein M Leeuwenburgh, Harm J T Rutten, Jacobus W A Burger, Johanne G Bloemen, Stijn H J Ketelaers, Usama Waqar, Tabish Chawla, Hareem Rauf, Pallavi Rani, Aaldert K Talsma, Lieke Scheurink, Jasper B van Praagh, Josefin Segelman, Jonas Nygren, Kajsa Anderin, Marit Tiefenthal, Beatriz de Andrés, Juan P Beltrán de Heredia, Andrea Vázquez, Tania Gómez, Parisa Golshani, Rawaz Kader, Abudi Mohamed, Marinke Westerterp, Andreas Marinelli, Quirine Niemer, Pascal G Doornebosch, Joël Shapiro, Maarten Vermaas, Eelco J R de Graaf, Hendrik L van Westreenen, Marije Zwakman, Annette D van Dalsen, Wouter J Vles, Joost Nonner, Boudewijn R Toorenvliet, Paul T J Janssen, Emiel G G Verdaasdonk, Femke J Amelung, Koen C M J Peeters Renu R Bahadoer, Fabian A Holman, Jeroen Heemskerk, Noortje Vosbeek, Jeroen W A Leijtens, Sophie B M Taverne, Bob H M Heijnen, Youssef El-Massoudi, Irene de Groot-van Veen, Christiaan Hoff, Daniela Jou-Valencia, Esther C J Consten Thijs A Burghgraef, Ritch Geitenbeek, Lorenzo G W L Hulshof, Gerrit D Slooter, Muriël Reudink, Nicole D Bouvy, Aurelia C L Wildeboer, Sonja Verstappen, Alexander J Pennings, Berber van den Hengel, Allard G Wijma, Jael de Haan, Lindsey C F de Nes, Vera Heesink, Tom Karsten, Charlotte M Heidsma, Willem J Koemans, Jan-Willem T Dekker, Charlène J van der Zijden, Daphne Roos, Ahmet Demirkiran, Sjirk van der Burg, Steven J Oosterling, Tijs J Hoogteijling, Bastiaan Wiering, Diederik P J Smeeing, Klaas Havenga, Hamid Lutfi, Esther C J Consten, Konstantinos Tsimogiannis, Filip Sköldberg, Joakim Folkesson, Frank den Boer, Ted G van Schaik, Pieter van Gerven, Colin Sietses, Jeroen C Hol, Evert-Jan G Boerma, Davy M J Creemers, Johannes K Schultz, Tone Frivold, Rolf Riis, Hilde Gregussen, Sondre Busund, Ole H Sjo, Maria Gaard, Nina Krohn, Amanda L Ersryd, Edmund Leung, Usama Waqar, Tabish Chawla, Hareem Rauf, Pallavi Rani, Hytham Sultan, Baraa Nabil Hajjaj, Ahmed Jehad Alhisi, Ahmed A E Khader, Ana Filipa Dias Mendes, Miguel Semião, Luis Queiroz Faria, Constança Azevedo, Helena M da Costa Devesa, Sónia Fortuna Martins, Aldo M Rodrigues Jarimba, Sónia M Ribeiro Marques, Rita Marques Ferreira, António Oliveira, Cátia Ferreira, Ricardo Pereira, Valeriu M Surlin, Giorgiana M Graure, Stefan Patrascu Sandu D Ramboiu, Ionut Negoi, Cezar Ciubotaru, Bogdan Stoica, Ioan Tanase, Bogdan Stoica, Cezar Ciubotaru, Valentina M Negoita, Sabrina Florea, Florin Macau, Mihai Vasile, Victor Stefanescu, Gabriel-Mihail Dimofte, Sorinel Luncă, Cristian-Ene Roată, Ana-Maria Mușină, Tatiana Garmanova, Mikhail N Agapov, Daniil G Markaryan, Galliamov Eduard, Alexey Yanishev, Alexander Abelevich, Andrey Bazaev, Sergey V Rodimov, Victor B Filimonov, Andrey A Melnikov, Igor A Suchkov, EvgeniyS Drozdov, Dmitriy N Kostromitskiy, Olle Sjöström, Peter Matthiessen, Bayar Baban, Soran Gadan, Kaveh Dehlaghi Jadid, Maria Staffan, Jennifer M Park, Daniel Rydbeck, Marie-Louise Lydrup, Pamela Buchwald, Henrik Jutesten, Lotten Darlin, Ebba Lindqvist, Karl Nilsson, Per-Anders Larsson, 186 Staffan Jangmalm, Jurij A Košir, Aleš Tomažič, Jan Grosek, Tajda Košir Božič, Aya Zazo, Rama Zazo, Hala Fares, Kusay Ayoub, Ammar Niazi, Ali Mansour, Ayman Abbas, Mohammad Tantoura, Alaa Hamdan, Naya Hassan, Bassam Hasan, Ahmad Saad, Amine Sebai, Anis Haddad, Houcine Maghrebi, Montasser Kacem, Ömer Yalkın, Mehmet Veysi Samsa, İbrahim Atak, Bengi Balci, Elifcan Haberal, Lütfi Dogan, Ibrahim E Gecim, Cihangir Akyol, Mehmet A Koc, Emre Sivrikoz, Deniz Piyadeoğlu, John O Larkin, Dara O avanagh, Selman Sökmen, Tayfun Bişgin, Erşan Günenç, Melek Güzel, Sezai Leventoğlu, Osman Yüksel, Ramazan Kozan, Hüseyin Göbüt, Fevzi Cengiz, Kemal Erdinc, Nihan Coşgun Acar, Erdinc Kamer, İlker Özgür, Oguzhan Aydın, Metin Keskin, Mehmet Türker Bulut, Cemil B Kulle, Yasin Kara, Osman Sıbıç, İbrahim H Özata, Dursun Buğra, Emre Balık, Cemil B Kulle, Murat Çakır, Anas Alhardan, Elif Colak, Ahmet B Ciftci, Engin Aybar, Ahmet Can Sari, Semra Demirli Atici, Tayfun Kaya, Ayberk Dursun, Bulent Calik, Ömer Faruk Özkan, Hanife Şeyda Ülgür, Özgül Düzgün, John Monson, Sarah George, Kayla Woods, Fatima Al-Eryani, Rudaina Albakry, Emile Coetzee, Adam Boutall, Ayesiga Herman, Claire Warden, Naser Mugla, Tim Forgan, Imraan Mia, Anton Lambrechts

**Affiliations:** Department of Surgery, Radboud University Medical Centre, Radboud Institute for Health Sciences, Nijmegen, The Netherlands; Department of Surgery, Radboud University Medical Centre, Radboud Institute for Health Sciences, Nijmegen, The Netherlands; Department of Surgery, Radboud University Medical Centre, Radboud Institute for Health Sciences, Nijmegen, The Netherlands; Department of Surgery, Amsterdam University Medical Centre, University of Amsterdam, Amsterdam, The Netherlands; Treatment and Quality of Life, Cancer Centre Amsterdam, Amsterdam, The Netherlands; Imaging and Biomarkers, Cancer Centre Amsterdam, Amsterdam, The Netherlands; Department of Surgery, Radboud University Medical Centre, Radboud Institute for Health Sciences, Nijmegen, The Netherlands; Department of Surgery, Canisius Wilhelmina Hospital, Nijmegen, The Netherlands; Department of Surgery, Amsterdam University Medical Centre, University of Amsterdam, Amsterdam, The Netherlands; Treatment and Quality of Life, Cancer Centre Amsterdam, Amsterdam, The Netherlands; Imaging and Biomarkers, Cancer Centre Amsterdam, Amsterdam, The Netherlands; Department of Medical Imaging, Radboud University Medical Centre, Radboud Institute for Health Sciences, Nijmegen, The Netherlands; Department of Surgery, UZ Leuven, Leuven, Belgium; Gastrointestinal Surgery Department, Hospital Clinic of Barcelona, University of Barcelona, Barcelona, Spain; Department of Digestive Surgery, Sorbonne Université, AP-HP, Hôpital Saint Antoine, Paris, France; Department of Surgery, University of Sydney Central Clinical School, Camperdown, New South Wales, Australia; Department of Surgery, Hospital La Fe, University of Valencia, Valencia, Spain; Department of Surgery, Hospital Alemán, Buenos Aires, Argentina; Bordeaux Colorectal Institute, Clinique Tivoli, Bordeaux, France; Colorectal Surgery, Hospital Alemão Oswaldo Cruz, São Paulo, Brazil; Department of Colon and Rectal Surgery, The University of Texas MD Anderson Cancer Center, Anderson, Texas, USA; Colorectal Surgery Centre, Groupe Hospitalier Privé Ambroise Paré-Hartmann, Neuilly Seine, France; Diagnostics and Intervention, Surgery, Umeå University, Umeå, Sweden; Department of Surgery, Amsterdam University Medical Centre, University of Amsterdam, Amsterdam, The Netherlands; Treatment and Quality of Life, Cancer Centre Amsterdam, Amsterdam, The Netherlands; Imaging and Biomarkers, Cancer Centre Amsterdam, Amsterdam, The Netherlands; Department of Surgery, Canisius Wilhelmina Hospital, Nijmegen, The Netherlands; Department of Surgery, Amsterdam University Medical Centre, University of Amsterdam, Amsterdam, The Netherlands; Treatment and Quality of Life, Cancer Centre Amsterdam, Amsterdam, The Netherlands; Imaging and Biomarkers, Cancer Centre Amsterdam, Amsterdam, The Netherlands; Department of Surgical Oncology and Gastrointestinal Surgery, Erasmus Medical Centre, Rotterdam, The Netherlands; Department of Surgery, Radboud University Medical Centre, Radboud Institute for Health Sciences, Nijmegen, The Netherlands

## Introduction

Colorectal-vaginal fistula (CRVF) is a challenging complication of rectal cancer resection, characterized by a leaking anastomosis in combination with vaginal discharge. Recent studies report incidences of CRVF after low anterior resection for rectal cancer between 1.6% and 5.1%, depending on the definition and the observation interval^1–3^ . Clinically, CRVF can present with (recurrent) vaginitis, faecal incontinence, and/or foul-smelling vaginal discharge due to passage of flatus or stool through the vagina^1^. CRVF can lead to chronic infections, multiple operations, sexual dysfunction, delayed adjuvant therapy, and a substantial reduction in quality of life^1,2^. Therefore, adequate management of CRVF is important to mitigate the negative consequences.

Management of CRVF is challenging due to the fact that the fistula remains a route of least resistance, prohibiting spontaneous healing in the presence of a competent internal sphincter. Management options for CRVF include conservative treatment (for example antibiotics), local endoscopic intervention (for example clipping, endosponge), local closure, and more invasive approaches, such as redo colorectal anastomosis, or dismantling or intersphincteric resection of the anastomosis with construction of an end colostomy^1,2,4–9^. In clinical practice, treatment decision-making is often influenced by several factors, such as clinical presentation, location and size of the fistula, and the quality of the colorectal anastomosis and surrounding tissue^1,4^ . Most studies reporting treatment of CRVF are limited by small cohorts and/or heterogeneity of the study population (for example also including patients after gynaecological resection)^1,10^.

Hence, the aim of this study was to examine the characteristics and treatment of patients with CRVF after rectal cancer resection in a large, international, multicentre database of patients with anastomotic leakage (AL), and to compare treatment and outcomes after AL in female patients without CRVF^11^.

## Methods

### Study design

The TENTACLE–Rectum study (TreatmENT of AnastomotiC LeakagE after rectal cancer resection) was an international, multicentre, retrospective cohort study involving 216 centres from 45 countries that included 2470 patients with AL after rectal cancer resection^4,12^. The TENTACLE–Rectum study was approved by the institutional review board of the Radboud University Medical Centre Nijmegen (file number 2009-5849)^4^. All collaborating centres adhered to the regulations of their national ethical committees. The study was registered at ClinicalTrials.gov (NCT04127734) and was conducted in agreement with the STROBE guidelines for reporting of observational studies^4,11,13^.

### Patient selection

Patients with rectal cancer operated between 1 January 2014 and 31 December 2018 were eligible for the TENTACLE–Rectum study if they were diagnosed with AL within 1 year after surgery. AL was defined, in accordance with an international consensus definition, as ‘a defect of the intestinal wall at the anastomotic site (including suture and staple lines of neorectal reservoirs) leading to a communication between the intra- and extraluminal compartments’^14^. Inclusion criteria were an age of ≥18 years, having an adenocarcinoma with its lower border below the level of the sigmoid take-off, and having undergone surgical resection with the creation of a primary anastomosis. The indication could be primary cancer, salvage resection for regrowth, or completion surgery after local excision^4^ . Exclusion criteria were benign disease, locally recurrent rectal cancer, or emergency surgery^4^. For the present analysis, all female patients with AL were selected and those with a CRVF were compared with those without a CRVF. CRVF was defined as a connection between a defect in the anastomosis and a defect in the vaginal wall. A reactivation AL was defined as AL diagnosed after the reversal of a primary or secondary diverting stoma to restore bowel continuity, due to failed healing of the anastomosis by primary or secondary intention^15^.

### Outcomes

The primary outcome was one-year stoma-free survival, defined as being alive without a temporary or permanent ileostomy or colostomy 1 year after rectal cancer resection. Secondary outcome measures were the number of reinterventions (endoscopic, radiological, surgical), postoperative day of first surgical intervention, total duration of hospital stay, intensive care unit (ICU) admission, total duration of ICU stay, and time to healing of AL. Treatment strategy and associated one-year stoma-free survival was separately analysed for patients who had a primary diverting stoma and those who did not.

### Statistical analysis

Dichotomous data were presented as *n* (%) and continuous data were presented as mean (s.d.) or median (interquartile range (i.q.r.)), as appropriate. The Pearson chi-squared test was used for the analysis of categorical variables and the Mann–Whitney *U* test was used for the analysis of continuous variables. *P* < 0.050 was considered statistically significant. Statistical analysis was performed using SPSS^®^ (IBM, Armonk, NY, USA; version 29).

## Results

### Baseline characteristics

Of 2470 patients with AL, 694 female patients were included, of whom a total of 88 (12.7%) presented with CRVF and 606 (87.3%) patients did not. No significant differences in age, ASA grade, BMI, and proportions of neoadjuvant therapy were found between the patients with CRVF and the patients without CRVF (*Table S1*).

Patients with CRVF more often had a lower tumour border from the anorectal junction (ARJ) (median of 43 mm *versus* 55 mm; *P* = 0.013). They more often underwent a multivisceral resection (MVR) (19.5% *versus* 10.3%; *P* = 0.011), especially vaginal resection (11 of 17 (6.5%) *versus* 6 of 61 (9.8%); *P* < 0.001), and they more often had a primary diverting stoma (72.7% *versus* 59.1%; *P* = 0.014). In the CRVF group, median time to AL diagnosis was longer (18 (i.q.r. 8–55) days *versus* 7 (i.q.r. 4–15) days; *P* < 0.001), abdominal contamination was less frequent (20.0% *versus* 42.5%; *P* < 0.001), and reactivation leakages were more often observed after reversal of a diverting stoma (32.1% *versus* 9.9%; *P* < 0.001) compared with patients without CRVF.

### Differences in treatment depending on the presence of CRVF

**Fig. 1:**
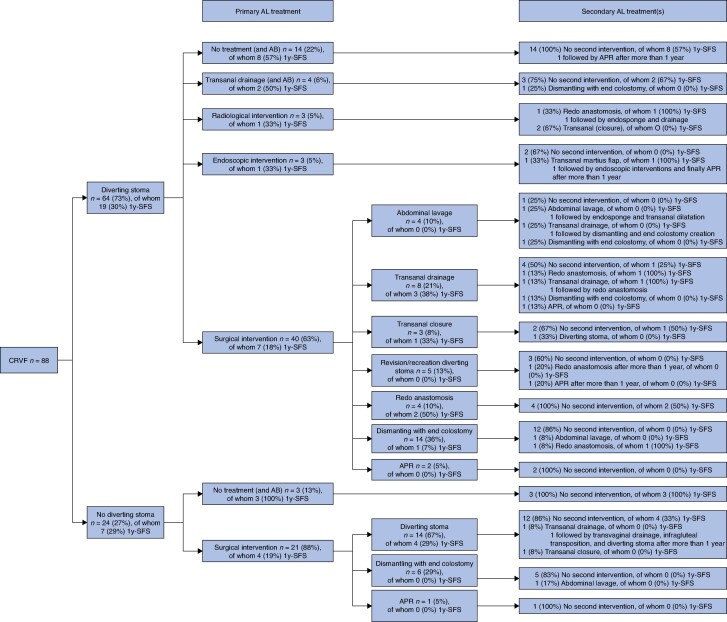
Patient flow diagram for treatment of CRVF Values are presented as frequency with corresponding percentage. Stoma interventions are not included and only primary-treatment endoscopic/radiological interventions are presented. CRVF, colorectal-vaginal fistula; 1y-SFS, 1-year stoma-free survival; AL, anastomotic leakage; AB, antibiotics; APR, abdominoperineal resection with proctectomy, resection of the anastomosis and end-colostomy.

Treatment of AL in patients with (or without) CRVF is outlined in *Table S2* and *Fig. 1*. Management in patients with CRVF was more often surgical compared with patients without CRVF (73.9% *versus* 54.3%; *P* < 0.001). The proportion of patients who underwent endoscopic interventions was similar in the CRVF group and the non-CRVF group (10.2% *versus* 10.7% respectively; *P* = 0.887), and radiological interventions were less common in the CRVF group (3.4% *versus* 12.0%; *P* = 0.015). The median time to the first surgical intervention was 22 (i.q.r. 10–166) days in patients with CRVF compared with 7 (i.q.r. 4–16) days in patients without CRVF (*P* < 0.001). In 88 patients with CRVF, the median total number of reinterventions was 148 and the median total number of surgical interventions was 136. The median total duration of hospital stay was shorter for patients with CRVF (10 days *versus* 15 days; *P* = 0.013).

### One-year stoma-free survival

One-year stoma-free survival was significantly lower in patients with CRVF compared with patients without CRVF (29.5% *versus* 48.7%; *P* = 0.002) (*Table S2*). A total of 64 (72.7%) patients with CRVF underwent a primary diverting stoma procedure (*Fig. 1*). Among those 64 patients with a diverted leak, conservative treatment of AL was initiated in 18 (28.1%) patients, with only antibiotics (14 patients) or transanal drainage on the ward (4 patients). One of those 18 patients underwent dismantling of the anastomosis and, eventually, 10 of 18 patients were without a stoma at 1 year. In the other 46 (71.9%) patients, initial treatment was surgical in 40 patients, endoscopic in 3 patients, and radiological in 3 patients. A redo anastomosis procedure was performed at some stage during treatment in 9 patients and 6 of those were without a stoma at 1 year. Eventually, the anastomosis was dismantled in 17 patients and a proctectomy with resection of the anastomosis (that is abdominoperineal resection (APR)) was performed in 5 patients.

A total of 24 patients with CRVF did not undergo a primary diverting stoma procedure (*Fig. 1*). Three patients were managed conservatively and all three were alive without a stoma at 1 year. Twenty-one patients were treated surgically, of whom 14 patients underwent a secondary diverting stoma procedure. None of those 21 patients underwent a redo anastomosis procedure at some stage during treatment. The anastomosis was dismantled in 6 patients and an APR was performed in 1 patient. Eventually, 4 of the surgically treated patients were alive without a stoma after 1 year and all of them underwent a secondary diverting stoma procedure.

### Stoma-free survival in CRVF patients

When comparing patients with CRVF who were stoma-free or not at 1 year, anastomotic defect circumference was smaller in patients with restored bowel continuity (0–25% of circumference in 12 of 15 (80.0%) *versus* 11 of 28 (39.3%); *P* = 0.011) (*Table S3*). The proportion of reactivation leakages was lower in stoma-free patients (4 of 24 (16.7%) *versus* 14 of 32 (43.8%); *P* = 0.032). Age, ASA, BMI, proportions of neoadjuvant therapy, abdominal approach, MVR, and the presence of a diverting stoma during rectal cancer resection were similar.

## Discussion

This large, international, multicentre, retrospective cohort study investigated characteristics, treatment, and outcomes of patients with CRVF after restorative rectal cancer resection. Index surgery in patients who developed CRVF more often included MVR (especially vaginal resection). CRVF patients more often had a primary diverting stoma, had a longer median time to AL diagnosis, less often had abdominal contamination, and more often had reactivation leakages. At 1 year after surgery, only three of ten were stoma-free compared with almost half of the patients without a CRVF. Twelve of 15 patients with CRVF who were stoma-free at 1 year had a small defect size (0–25% of circumference), while this was much lower in patients with a stoma at 1 year. Remarkably, several patient factors (for example age, ASA), neoadjuvant radiotherapy, and the presence of a diverting stoma did not seem to be associated with stoma-free survival among patients with CRVF.

What are the potential explanations for the worse prognosis of CRVF? If compared with female patients with AL but without CRVF, there were no differences in age, ASA, or BMI, while these were predictive factors in the previously published STOMA score^12^. Therefore, it seems that the low one-year stoma-free survival rate cannot be attributed to patient factors. Regarding index surgery, there was even a slightly higher proportion of full splenic flexure mobilization in the CRVF group, corresponding with a mean shorter distance of the anastomosis to the ARJ. This makes tension on the anastomosis an unlikely explanation. However, significantly more MVR was performed, which might have contributed to the lower stoma-free survival. The anastomosis was more often in a side-to-end configuration in CRVF patients, although this is not a known risk factor. The proportion of patients with a primary diverting stoma was higher in the CRVF group, while it is often stated that defunctioning can help in preservation of the anastomosis. Similarly, a contradictory trend was observed regarding quick sequential organ failure assessment (qSOFA) scores, showing that patients with CRVF seemed to be even less sick.

Among patients with CRVF, patient factors (age, ASA, BMI) and the proportion of primary diversion were also not different between those with or without a stoma at 1 year. Remarkably, the proportions of neoadjuvant radiotherapy were also not different. The only clear difference was the defect circumference, with the smallest defects (0–25% of circumference) being significantly over-represented in the patients who were stoma-free afterat 1 year, which is in line with the literature^6^. None of the patients with a defect circumference beyond 50% was stoma-free after 1 year.

The low stoma-free survival rate in patients who develop CRVF is most likely related to pathophysiology. If an anastomotic leak is draining to the vagina, this is a route of least resistance compared with a competent anal sphincter. With such a drainage route, there will be less pus accumulation under pressure in the perianastomotic abscess, which likely explains the relatively low qSOFA scores and the lower rate of abdominal contamination. While this has an initial advantage, the vaginal drainage likely prohibits healing of the leak afterwards. Without surgical repair, a fistula tract with a short distance between the bowel and the vagina becomes epithelialized and is unlikely to heal spontaneously. After repair of the bowel and vagina, the competent anal sphincter can again cause a recurrent fistula. Even when repair seemed to be successful with subsequent stoma reversal, there was a high proportion of reactivation leakage in the CRVF group. Restoration of bowel continuity puts pressure on a fragile healed anastomosis, with seemingly a high risk of recurrent fistula.

Remarkably, the time to AL diagnosis was significantly longer in the CRVF group. It seems that patients do not easily relate vaginal discharge to a leak. Furthermore, 72.7% of these leaks were defunctioned, which minimizes discharge and symptoms. Surgeons should probably be more proactive in diagnosing leaks in patients with CRVF, especially in patients undergoing MVR including the posterior vaginal wall, although it remains unknown if early surgical repair can improve success rates.

What can be learned from this observational cohort data? First, patients with ‘limited’ leaks with CRVF who are candidates for conservative management have a relatively high chance of ultimately having bowel continuity restored. Accurate assessment of defect size is difficult, but a registered defect size ≤25% likely corresponds to a ‘pinhole’ leak in most patients. A redo anastomosis procedure in selected patients can be successful in a similar proportion. This has been recently demonstrated in video presentations with various techniques such as single stapled anastomosis or a pull-through technique^16–18^. Other techniques, such as the use of bioprosthetics, are uncommonly used in patients after rectal cancer surgery, so these do not seem to play an important role^19^. Defect circumference seems to be the main driver for becoming stoma-free. Therefore, surgeons should probably be reserved in their attempts to restore bowel continuity in patients with an a priori low likelihood of success based on this leakage characteristic. Dismantling can then be a good option, although the rectal stump might still maintain an active fistula to the vagina, with significant discharge and compromised quality of life. Therefore, proctectomy and filling of the pelvis (using, for example, omentoplasty), although not effective in primary APR, should be considered as an alternative in these patients^20^.

The major strength of this study is the robust and detailed data of a large multicentre and international population, while other studies were impeded by small sample sizes and heterogeneous populations (for example also benign disease) and relatively short follow-up^10,21–23^. Limitations are related to the retrospective design, which could have contributed to data inconsistencies and missing data, but intensive data cleaning, verification, and validation was performed to mitigate this limitation^4^. Furthermore, the TENTACLE–Rectum study was not specifically designed to analyse CRVF and CRVF was not defined according to a generally accepted definition, as this remains lacking. However, the current definition was in line with how data were consistently entered into the database, thereby promoting validity and homogeneity in the reporting of CRVF.

The female patients who are collected in this study were all patients who were entered into the TENTACLE–Rectum AL database^11^. This means that patients might have been missed, such as patients with a CRVF documented as ‘fistula’ in the electronic patient file rather than ‘AL’, patients with only a minor CRVF without septic symptoms, or those who develop CRVF beyond 1 year after index resection (for example after late stoma closure). This might have influenced the observed low stoma-free survival in this study, as some patients with potentially better outcomes were not included. Unfortunately, specific characteristics of the vaginal fistula (for example exact location, defect size, extent of discharge) were not collected, but the reliability of assessing such criteria in daily practice and their clinical relevance can be questioned. Furthermore, data regarding the (potential) aetiology of CRVF were lacking, for instance whether the posterior vaginal wall was included in the anastomosis during circular stapling. Moreover, reconstruction techniques as described in CRVF not related to rectal cancer were not specifically scored in the database and only recorded in free-text remarks. These techniques, such as Martius plasty, gracilis muscle interposition, and pull-through coloanal anastomosis, are all described in expert centres with higher success rates^24,25^. However, only a minority of patients have undergone rectal cancer surgery as the reason for CRVF in these expert series. This is probably due to the fact that the rectum, including the mesorectum, is (nearly) completely removed, making such reconstructions more difficult compared with CRVF after trauma or post-partum. Finally, the proportion of APR as definitive treatment might be underestimated, as this was based on free-text remarks in the database and some patients even underwent APR after 1 year and, as such, the stoma-free survival was perhaps even lower than reported.

In conclusion, female patients developing AL with CRVF after rectal cancer resection are unlikely to become stoma-free, and the majority require surgical reintervention(s), even in the presence of a primary diverting stoma. These data from a large international database can be used for adequate patient counseling in women who present with CRVF. Patients with small leaks that can be managed conservatively, and those being candidates for a redo anastomosis have a reasonable chance for ultimate bowel continuity.

## Collaborators

Andreas J.A. Bremers, Floris T. Ferenschild (Radboud University Medical Centre, Radboud Institute for Health Sciences, Nijmegen, The Netherlands); Stefanie de Vriendt, André D’Hoore, Gabriele Bislenghi (University Hospitals Leuven, Leuven, Belgium); Jordi Farguell, Antonio M. Lacy, Paula González Atienza (Hospital Clínic de Barcelona, Barcelona, Spain); Charlotte S. van Kessel (Royal Prince Albert Hospital, Sydney, Australia); Yann Parc, Thibault Voron, Maxime K. Collard (Sorbonne Université, AP-HP, Hôpital Saint Antoine, Paris, France); Jorge Sancho Muriel, Hannia Cholewa (Valencia University Hospital La Fe, Valencia, Spain); Laura A. Mattioni (Hospital Alemán, Buenos Aires, Argentina); Alice Frontali (Beaujon Hospital, Clichy, and University of Paris, Clichy, France); Sebastiaan W. Polle, Fatih Polat, Ndidi J. Obihara (Canisius Wilhelmina Hospital, Nijmegen, The Netherlands); Bruna B. Vailati (Hospital Alemão Oswaldo Cruz, São Paulo, Brazil); Miranda Kusters, Jurriaan B. Tuynmann, Sanne J.A. Hazen, Alexander A.J. Grüter (Amsterdam University Medical Centres, location VUmc, Amsterdam, The Netherlands; Cancer Centre Amsterdam, Treatment and Quality of Life, Amsterdam, The Netherlands; Cancer Centre Amsterdam, Imaging and Biomarkers, Amsterdam, The Netherlands); Takahiro Amano, Hajime Fujiwara (Cancer Institute Hospital of the Japanese Foundation for Cancer Research, Tokyo, Japan); Mario Salomon, Hernán Ruiz, Ricardo Gonzalez, Diego Estefanía (Buenos Aires British Hospital, Buenos Aires, Argentina); Nicolas Avellaneda, Augusto Carrie, Mateo Santillan (CEMIC University Hospital, Buenos Aires, Argentina); Diana A. Pantoja Pachajoa, Matias Parodi, Manuel Gielis (Clínica Universitaria Reina Fabiola, Córdoba, Argentina); Alf-Dorian Binder, Thomas Gürtler, Peter Riedl (Universitätsklinikum Tulln, Tulln an der Donau, Austria); Sarit Badiani, Christophe Berney, Matthew Morgan (Bankstown-Lidcombe Hospital, Sydney, New South Wales, Australia); Paul Hollington, Nigel da Silva, Gavin Nair (Flinders Medical Centre, Adelaide, South Australia, Australia); Yiu M. Ho, Michael Lamparelli, Raj Kapadia (Rockhampton Hospital, Queensland, Australia); 19 Hidde M. Kroon, Nagendra N. Dudi-Venkata, Jianliang Liu, Tarik Sammour (Royal Adelaide Hospital, Adelaide, South Australia, Australia); Nicolas Flamey, Paul Pattyn, Ahmed Chaoui, Louis Vansteenbrugge (AZ Delta, Roeselare, Belgium); Nathalie E.J. van den Broek, Patrick Vanclooster, Charles de Gheldere (Heilig-Hartziekenhuis, Lier, Belgium); Pieter Pletinckx, Barbara Defoort, Maxime Dewulf (Maria Middelares Ghent, Belgium); Mihail Slavchev, Nikolay Belev, Boyko Atanasov, Panche Krastev (University Hospital Eurohospital—Medical University Plovdiv, Plovdiv, Bulgaria); Manol Sokolov, Svilen Maslyankov, Petar Gribnev, Vasil Pavlov (Aleksandrovska University Hospital, Sofia, Bulgaria); Tsvetomir Ivanov, Martin Karamanliev, Emil Filipov, Pencho Tonchev (Medical University Pleven, Pleven, Bulgaria); Felix Aigner, Martin Mitteregger, Caterina Allmer, Gerald Seitinger (St. John of God Hospital Graz, Graz, Austria); Nicola Colucci, Nicolas Buchs, Frédéric Ris, Christian Toso (Geneva University Hospitals and Faculty of Medicine, Geneva, Switzerland); Eleftherios Gialamas, Aurélie Vuagniaux, Roland Chautems, Marc-Olivier Sauvain (Neuchâtel Hospital, Neuchâtel, Switzerlandl); Silvio Daester, Markus von Flüe, Marc-Olivier Guenin, Stephanie Taha-Mehlitz, Gabriel F. Hess (St. Clara Hospital and University Hospital Basel, Basel, Switzerland); Lubomír Martínek, Matej Skrovina, Maria Machackova, Vladimir Benčurik (Hospital Nový Jičín, Nový Jičín, Czech Republic); Deniz Uluk, Johann Pratschke, Luca S. Dittrich, Safak Guel-Klein (Charité-Universitätsmedizin Berlin, Corporate Member of Freie Universität Berlin and Humboldt-Universität zu Berlin and Berlin Institut of Health, Berlin, Germany); Daniel Perez (Asclepios Clinic Altona, Hamburg, Germany); Julia-Kristin Grass, Nathaniel Melling, Simone Mueller (University Medical Centre of Hamburg-Eppendorf, Hamburg, Germany); Lene H. Iversen, Jacob D. Eriksen (Aarhus University Hospital, Aarhus, Denmark); Gunnar Baatrup, Issam Al-Najami, Thomas Bjørsum-Meyer (Odense University Hospital, Svendborg Sygehus, Denmark); Jüri Teras, Roland M. Teras (North Estonia Medical Centre Foundation, Tallinn, Estonia); Fatma A. Monib, Nagm Eldin Abu Elnga Ahmed, Eithar Alkady, Ahmed K. Ali (Assiut University Hospital, Assiut, Egypt); Gehan Abd Elatti Khedr, Ahmed Samir Abdelaal, Fouad M. Bassyouni Ashoush, Moataz Ewedah (Alexandria Main University Hospital, Alexandria Governorate, Egypt); Eslam M. Elshennawy, Mohamed Hussein (Kafr Elshikh University Hospital, Kafr el-Sheikh, Egypt); Daniel Fernández-Martínez, Luis J. García-Flórez, María Fernández-Hevia, Aida Suárez-Sánchez (Central University Hospital of Asturias, Asturias, Spain); Izaskun del Hoyo Aretxabala, Iria Losada Docampo, Jesús Gómez Zabala (Basurto University Hospital, Bilbao, Spain); Patricia Tejedor, Javier T. Morales Bernaldo de Quirós, Ignacio Bodega Quiroga (Hospital Universitario Gómez Ulla, Spain); Antonio Navarro-Sánchez, Iván Soto Darias, Cristina López Fernández, Cristina de La Cruz Cuadrado (Hospital Materno Infantil de Gran Canaria, Las Palmas, Spain); Luis Sánchez-Guillén, Francisco López-Rodríguez-Arias, Álvaro Soler-Silva, Antonio Arroyo (University Hospital of Elche, Elche, Spain); Juan C. Bernal-Sprekelsen, Segundo Á. Gómez-Abril, Paula Gonzálvez, María T. Torres (Hospital Universitario Dr. Peset, Valencia, Spain); Teresa Rubio Sánchez, Francisco Blanco Antona, Juan E. Sánchez Lara, José A. Alcázar Montero (University Hospital of Salamanca, Salamanca, Spain); Fernando Mendoza-Moreno, Manuel Díez-Alonso, Belén Matías-García, Ana Quiroga-Valcárcel (Hospital Universitario Príncipe de Asturias, Spain); Enrique Colás-Ruiz, Marta M. Tasende-Presedo, Ignacio Fernández-Hurtado, José A. Cifuentes-Ródenas, Marta Castro Suárez (Son Llàtzer Hospital, Illes Balears, Spain); Manuel Losada, Miguel Hernández, Alfredo Alonso, Beatriz Diéguez (Hospital Universitario del Sureste, Madrid, Spain); Daniel Serralta, Rita E. Medina Quintana, Jose M. Gil Lopez, Francisca Lima Pinto, Elena Nieto-Moreno (Hospital Infanta Leonor, San Sebastián de los Reyes, Madrid, Spain); Alba Correa Bonito, Carlos Cerdán Santacruz, Elena Bermejo Marcos, Javier García Septiem (University Hospital de La Princesa, Madrid, Spain); Aránzazu Calero-Lillo, Javier Alanez-Saavedra, Salvador Muñoz-Collado, Manuel López-Lara (Fundación Hospital del Espíritu Santo, Santa Coloma de Gramenet, Barcelona, Spain); María Labalde Martínez, Eduardo Ferrero Herrero, Francisco Javier García Borda, Óscar García Villar (12 de Octubre University Hospital, Madrid, Spain); Jorge Escartín, Juan L. Blas, Rocío Ferrer, Jorge García Egea (Hospital Royo Villanova, Zaragoza, Spain); Antonio Rodríguez-Infante, Germán Mínguez-Ruiz, Guillermo Carreño-Villarreal, Gerardo Pire-Abaitua (Hospital Universitario San Agustín, Avilés, Spain); Jana Dziakova, Carlos Sáez-Cazallas Rodríguez, María J. Pizarro Aranda, José M. Muguerza Huguet (Hospital Universitario Clínico San Carlos, Madrid, Spain); Nerea Borda-Arrizabalaga, José M. Enriquez-Navascués, Garazi Elorza Echaniz, Yolanda Saralegui Ansorena (Donostia University Hospital, Donostia, Spain); Mercedes Estaire-Gómez, Carlos Martínez-Pinedo, Alejandro Barbero-Valenzuela, Pablo Ruíz-García (Hospital General Universitario de Ciudad Real, Ciudad Real, Spain); Miquel Kraft, María J. Gómez-Jurado, Gianluca Pellino, Eloy Espín-Basany (Vall d'Hebron University Hospital, Universitat Autonoma de Barcelona, Barcelona, Spain); Eddy Cotte, Nathalie Panel, Claire-Angéline Goutard (Hospices Civils de Lyon, Lyon Sud University Hospital, Pierre Bénite, France); Nicola deÁngelis, Lelde Lauka (Henri Mondor Hospital, AP-HP, Créteil, France); Shafaque Shaikh, Laura Osborne, George Ramsay (Aberdeen Royal Infirmary, NHS Grampian, Aberdeen, UK); Vladimir-Ion Nichita, Santosh Bhandari, Panchali Sarmah (Cambridgeshire in Peterborough City Hospital, Peterborough, UK); Rob M. Bethune, Heather C.M. Pringle, Lisa Massey, George E. Fowler (Royal Devon and Exeter Hospital, Exeter, UK); Hytham K.S. Hamid, Belinda D. de Simone (East Kent Hospitals University NHS Foundation Trust, Ashford, UK); James Kynaston, Nicholas Bradley, Roxane M. Stienstra (Forth Valley Royal Hospital, Larbert, Scotland); Shashank Gurjar, Tanmoy Mukherjee, Ashfaq Chandio, Safia Ahmed (Bedfordshire Hospitals NHS Foundation Trust, Luton, UK); Baljit Singh, Francois Runau, Sanjay Chaudhri, Oliver Siaw (Leicester General Hospital, Leicester, UK); Janahan Sarveswaran, Victor Miu, Daniel Ashmore, Haitham Darwich (Pinderfields Hospital, Wakefield, UK); Deepak Singh-Ranger, Nirbhaibir Singh (The Royal Wolverhampton NHS Trust, Wolverhampton, West Midlands, UK); Mohamed Shaban (Newcastle upon Tyne NHS Foundation Trust, Newcastle upon Tyne, UK); Fahed Gareb (Queen Elizabeth The Queen Mother Hospital, Margate, UK); Thalia Petropolou, Adreas Polydorou (Euroclinic Athens, Athens, Greece); Mit Dattani, Asma Afzal (University Hospitals Birmingham NHS Foundation Trust, Birmingham, UK); Akshay Bavikatte, Boby Sebastian, Nicholas Ward, Amitabh Mishra (West Suffolk Hospital, Suffolk, UK); Dimitrios Manatakis, Christos Agalianos,Nikolaos Tasis, Maria-Ioanna Antonopoulou (Athens Naval and Veterans Hospital, Athens, Greece); Ioannis Karavokyros, Alexandros Charalabopoulos, Dimitrios Schizas, Efstratia Baili, Athanasios Syllaios, Lysandros Karydakis, Michail Vailas (Laikon General Hospital- National and Kapodistrian University of Athens, Greece); Dimitrios Balalis, Dimitrios Korkolis, Aris Plastiras, Aliki Rompou (Saint Savvas Anti-Cancer Hospital, Athens Greece); Sofia Xenaki, Evangelos Xynos, Emmanuel Chrysos, Maria Venianaki (University Hospital of Heraklion Crete, Greece); Grigorios Christodoulidis, Konstantinos Perivoliotis, George Tzovaras, Ioannis Baloyiannis (University Hospital of Larissa, Larissa Greece); Man-Fung Ho, Simon Siu-man Ng, Tony Wing-chung Mak, Kaori Futaba (Prince of Wales Hospital, The Chinese University of Hong Kong, Shatin, Hong Kong); Goran Šantak, Damir Šimleša, Jurica Ćosić, Goran Zukanović (General County Hospital Požega, Požega, Croatia); Michael E. Kelly, John O. Larkin, Paul H. McCormick, Brian J. Mehigan (The Trinity St. James’s Cancer Institute, Dublin + School of Medicine, Trinity College Dublin, Ireland); Tara M. Connelly, Peter Neary, Jessica Ryan, Peter McCullough (University Hospital Waterford, Waterford, Ireland); Maytham A. Al-Juaifari, Hayder Hammoodi, Ali Hashim Abbood (Al-Sadder Teaching Hospital, Najaf, Iraq); Marcello Calabrò, Andrea Muratore, Antonio La Terra, Francesca Farnesi (Edoardo Agnelli Hospital, Pinerolo, Italy); Carlo V. Feo, Nicolò Fabbri, Antonio Pesce, Marta Fazzin (Azienda Unità Sanitaria Locale di Ferrara, Università di Ferrara, Ferrara, Italy); Francesco Roscio, Federico Clerici (ASST Valle Olona Busto Arsizio Italy, Busto Arsizio VA, Italy); Andrea Lucchi, Laura Vittori, Laura Agostinelli, Maria Cristina Ripoli (AUSL Romagna Ceccarini Hospital, Riccione, Italy); Daniele Sambucci, Andrea Porta (Fatebenefratelli Hospital ‘Holy Family’, Erba, Italy); Giovanni Sinibaldi, Giacomo Crescentini, Antonella larcinese, Emanuele Picone (Fatebbenefratelli Hospital, Isola Tiberina, Rome, Italy); Roberto Persiani, Alberto Biondi, Roberto Pezzuto, Laura Lorenzon, Gianluca Rizzo, Claudio Coco, Luca D’Agostino (‘A. Gemelli’ University Hospital, Catholic University of Rome, Rome, Italy); Antonino Spinelli, Matteo M. Sacchi, Michele Carvello, Caterina Foppa (Humanitas University, Milan, Italy); Antonino Spinelli, Matteo M. Sacchi, Michele Carvello, Caterina Foppa, Annalisa Maroli (IRCCS Humanitas Research Hospital, Milan, Italy); Gian M. Palini, Gianluca Garulli, Nicola Zanini (Infermi Hospital of Rimini, AUSL Della Romagna, Rimini, Italy); Paolo Delrio, Daniela Rega, Fabio Carbone, Alessia Aversano (Fondazione Giovanni Pascale—IRCCS, Naples, Italy); Giovanni Pirozzolo, Alfonso Recordare, Lucrezia D'Alimonte, Chiara Vignotto (Dell'Angelo Hospital, Venice, Italy); Carlo Corbellini, Gianluca M. Sampietro, Leonardo Lorusso, Carlo A. Manzo (ASST Rhodense, Rho Memorial Hospital, Milano, Italy); Federico Ghignone, Giampaolo Ugolini, Isacco Montroni, Franceso Pasini (Ospedale Santa Maria delle Croci, Ravenna, Italy); Francesco Pasini (Ospedale per gli Infermi, Faenza, Italy); Michele Ballabio, Pietro Bisagni, Francesca T. Armao, Marco Longhi (Maggiore Hospital in Lodi, Lodi, Italy); Omar Ghazouani, Raffaele Galleano (Santa Corona Hospital, Pietra Ligure, Italy); Nicolò Tamini, Massimo Oldani, Luca Nespoli (San Gerardo Hospital, Monza, Italy); Arcangelo Picciariello, Donato F. Altomare, Giovanni Tomasicchio, Giuliano Lantone (University of Bari Aldo Moro, Bari, Italy); Fausto Catena, Mario Giuffrida, Alfredo Annicchiarico, Gennaro Perrone (Parma University Hospital, Parma, Italy); Ugo Grossi, Giulio A. Santoro, Giacomo Zanus, Alessandro Iacomino, Simone Novello, Nicola Passuello, Martino Zucchella (Regional Hospital Treviso, Treviso, Italy); Lucia Puca, Maurizio deGiuli, Rossella Reddavid (San Luigi University Hospital, Orbassano, Torino, Italy); Stefano Scabini, Alessandra Aprile, Domenico Soriero, Emanuela Fioravanti (AOU San Martino Hospital, Genoa, Italy); Matteo Rottoli, Angela Romano, Marta Tanzanu, Angela Belvedere (IRCCS Azienda Ospedaliero Universitaria di Bologna, Bologna, Italy); Nicolò M. Mariani, Andrea P. Ceretti, Enrico Opocher (ASST Santi Paolo e Carlo, Milan, Italy); Gaetano Gallo, Giuseppe Sammarco (University of Catanzaro, Catanzaro, Italy); Gilda de Paola (University of Milano, Milano, Italy); Salvatore Pucciarelli, Francesco Marchegiani, Gaya Spolverato, Gianluca Buzzi (Azienda Ospedale-Università di Padova, Padova, Italy); Salomone Di Saverio, Paola Meroni, Cristiano Parise, Elisa I. Bottazzoli (University of Insubria, University Hospital of Varese, ASST Sette Laghi, Regione Lombardia, Varese, Italy); Pierfrancesco Lapolla, Gioia Brachini, Bruno Cirillo, Andrea Mingoli (‘P. Valdoni’, Policlinico Umberto I University Hospital, Sapienza University of Rome, Rome, Italy); Giuseppe Sica, Leandro Siragusa, Vittoria Bellato, Daniele Cerbo (University of Rome ‘Tor Vergata’, Rome, Italy); Carlo A. de Pasqual, Giovanni de Manzoni, Maria A. di Cosmo (University of Verona, Verona, Italy); Bourhan M.H. Alrayes, Mahmoud W. M. Qandeel (Islamic Hospital Amman, Amman, Jordan); Mohammad Bani Hani (King Abdullah University Hospital, Ar-Ramtha, Jordan); Alexander Rabadi, Mohammad S. el Muhtaseb, Basel Abdeen, Fahed Karmi (The University of Jordan, Amman, Jordan); Justas Žilinskas, Tadas Latkauskas, Algimantas Tamelis, Ingrida Pikūnienė, Vygintas Šlenfuktas (Hospital of Lithuanian University of Health Sciences Kaunas Clinics, Kaunas, Lithuania); Tomas Poskus, Marius Kryzauskas, Matas Jakubauskas, Saulius Mikalauskas, Lina Jakubauskiene (Vilnius University, Vilnius, Lithuania); Soha Y. Hassan, Amani Altrabulsi (Benghazi Medical Centre, Benghazi, Libya); Eman Abdulwahed, Reem Ghmagh, Abdulqudus Deeknah, Entisar Alshareea (Tripoli Central Hospital, Tripoli, Libya); Muhammed Elhadi, Saleh Abujamra, Ahmed A. Msherghi, Osama W.E. Tababa (Tripoli University Hospital, Tripoli, Libya); Mohammed A. Majbar, Amine Souadka, Amine Benkabbou, Raouf Mohsine, Sabrillah Echiguer (National Institute of Oncology, University Mohammed V in Rabat, Rabat, Morocco); Paulina Moctezuma-Velázquez, Noel Salgado-Nesme, Omar Vergara-Fernández, Juan C. Sainz-Hernández, Francisco E. Alvarez-Bautista (Instituto Nacional de Ciencias Médicas y Nutrición Salvador Zubirán, Mexico City, Mexico); Andee D. Zakaria, Zaidi Zakaria, Michael P.K. Wong, Razif Ismail (Universiti Sains Malaysia, Kubang Kerian, Kelantan, Malaysia); Aini F. Ibrahim, Nik A.N. Abdullah, Rokayah Julaihi (Universiti Malaysia Sarawak, Kota Samarahan, Sarawak); Sameer Bhat, Greg O'Grady, Ian Bissett (University of Auckland, Auckland, New Zealand); Bas Lamme, Gijsbert D. Musters, Anne M. Dinaux (Albert Schweitzer Hospital, Dordrecht, The Netherlands); Brechtje A. Grotenhuis, Ernst J. Steller Arend G.J. Aalbers, Marjolein M. Leeuwenburgh (Netherlands Cancer Institute-Antoni van Leeuwenhoek, Amsterdam, The Netherlands); Harm J.T. Rutten, Jacobus W.A. Burger, Johanne G. Bloemen, Stijn H.J. Ketelaers (Catharina Hospital, Eindhoven, The Netherlands); Usama Waqar, Tabish Chawla, Hareem Rauf, Pallavi Rani (Aga Khan University, Karachi City, Pakistan); Aaldert K. Talsma, Lieke Scheurink, Jasper B. van Praagh (Deventer Hospital, Deventer, The Netherlands); Josefin Segelman, Jonas Nygren, Kajsa Anderin, Marit Tiefenthal (Ersta Hospital, Stockholm, Sweden); Beatriz de Andrés, Juan P. Beltrán de Heredia, Andrea Vázquez, Tania Gómez (University Clinical Hospital of Valladolid, Valladolid, Spain); Parisa Golshani, Rawaz Kader, Abudi Mohamed (Gävle Hospital, Gävle, Sweden); Marinke Westerterp, Andreas Marinelli, Quirine Niemer (Medical Centre Haaglanden, Westeinde, Den Haag, The Netherlands); Pascal G. Doornebosch, Joël Shapiro, Maarten Vermaas, Eelco J.R. de Graaf (Jsselland Hospital, Capelle Aan Den IJssel, The Netherlands); Hendrik L. van Westreenen, Marije Zwakman, Annette D. van Dalsen (Isala Hospital, Zwolle, The Netherlands); Wouter J. Vles, Joost Nonner, Boudewijn R. Toorenvliet, Paul T.J. Janssen (Ikazia Hospital, Rotterdam, The Netherlands); Emiel G.G. Verdaasdonk, Femke J. Amelung (Jeroen Bosch Hospital, ‘s-Hertogenbosch, The Netherlands); Koen C.M.J. Peeters Renu R. Bahadoer, Fabian A. Holman (Leiden University Medical Centre, Leiden, The Netherlands); Jeroen Heemskerk, Noortje Vosbeek, Jeroen W.A. Leijtens, Sophie B.M. Taverne (Laurentius Hospital, Roermond, The Netherlands); Bob H.M. Heijnen, Youssef El-Massoudi, Irene de Groot-van Veen (LangeLand Hospital, Zoetermeer, The Netherlands); Christiaan Hoff, Daniela Jou-Valencia (Medical Centre Leeuwarden, Leeuwarden, The Netherlands); Esther C.J. Consten Thijs A. Burghgraef, Ritch Geitenbeek, Lorenzo G.W.L. Hulshof (Meander Medical Centre, Amersfoort, The Netherlands); Gerrit D. Slooter, Muriël Reudink (Máxima Medical Centre, Veldhoven, The Netherlands); Nicole D. Bouvy, Aurelia C. L. Wildeboer, Sonja Verstappen, Alexander J. Pennings (Maastricht University Medical Centre, Maastricht, The Netherlands); Berber van den Hengel, Allard G. Wijma, Jael de Haan (Martini Hospital, Groningen, The Netherlands); Lindsey C.F. de Nes, Vera Heesink (Maasziekenhuis Pantein, Boxmeer, The Netherlands); Tom Karsten, Charlotte M. Heidsma, Willem J. Koemans (Onze Lieve Vrouwe Gasthuis, Amsterdam, The Netherlands); Jan-Willem T. Dekker, Charlène J. van der Zijden, Daphne Roos (Reinier de Graaf Gasthuis, Delft, The Netherlands); Ahmet Demirkiran, Sjirk van der Burg (Red Cross Hospital, Beverwijk, The Netherlands); Steven J. Oosterling, Tijs J. Hoogteijling (Spaarne Gasthuis, Haarlem, The Netherlands); Bastiaan Wiering, Diederik P.J. Smeeing (Slingeland Ziekenhuis, Doetinchem, The Netherlands); Klaas Havenga, Hamid Lutfi, Esther C.J. Consten (University Medical Centre Groningen, Groningen, The Netherlands); Konstantinos Tsimogiannis, Filip Sköldberg, Joakim Folkesson (Uppsala University, Uppsala, Sweden); Frank den Boer, Ted G. van Schaik, Pieter van Gerven (Zaans Medical Centre, Zaandam, The Netherlands); Colin Sietses, Jeroen C. Hol (Gelderse Vallei Hospital Ede, Ede, The Netherlands); Evert-Jan G. Boerma, Davy M.J. Creemers (Zuyderland Medical Centre, Sittard/Heerlen, The Netherlands); Johannes K. Schultz, Tone Frivold, Rolf Riis (Akershus University Hospital, Lørenskog, Norway); Hilde Gregussen, Sondre Busund (Hospital innland Hamar, Hamar, Norway); Ole H. Sjo, Maria Gaard, Nina Krohn, Amanda L. Ersryd (Ullevål Oslo University Hospital, Oslo, Norway); Edmund Leung (Hereford County Hospital, Hereford, UK); Usama Waqar, Tabish Chawla, Hareem Rauf, Pallavi Rani (Aga Khan University, Karachi City, Pakistan); Hytham Sultan, Baraa Nabil Hajjaj, Ahmed Jehad Alhisi, Ahmed A.E. Khader (Al-Shifa Hospital, Gaza City, Palestine); Ana Filipa Dias Mendes, Miguel Semião, Luis Queiroz Faria, Constança Azevedo (Centro Hospitalar Universitário Cova da Beira, Covilha, Portugal); Helena M. da Costa Devesa, Sónia Fortuna Martins, Aldo M. Rodrigues Jarimba, Sónia M. Ribeiro Marques (Hospital Distrital de Santarém, Santarém, Portugal); Rita Marques Ferreira, António Oliveira, Cátia Ferreira, Ricardo Pereira (Centro Hospitalar de Trás-os-Montes e Alto Douro EPE, Vila Real, Portugal); Valeriu M. Surlin, Giorgiana M. Graure, Stefan Patrascu Sandu D. Ramboiu (Clinical County Emergency Hospital of Craiova, University of Medicine and Pharmacy of Craiova, Romania); Ionut Negoi, Cezar Ciubotaru, Bogdan Stoica, Ioan Tanase (Carol Davila University of Medicine and Pharmacy Bucharest, Bucharest, Romania); Bogdan Stoica, Cezar Ciubotaru, Valentina M. Negoita (Clinical Emergency Hospital Bucharest, Bucharest, Romania); Sabrina Florea, Florin Macau, Mihai Vasile, Victor Stefanescu (Central Military Emergency Hospital Dr. Carol Davila, Bucharest, Romania); Gabriel-Mihail Dimofte, Sorinel Luncă, Cristian-Ene Roată, Ana-Maria Mușină (Regional Oncology Institute, Iasi, Romania); Tatiana Garmanova, Mikhail N. Agapov, Daniil G. Markaryan, Galliamov Eduard (Lomonosov Moscow State University, Moscow, Russia); Alexey Yanishev, Alexander Abelevich, Andrey Bazaev (Privolzhsky Research Medical University, Nizhny Novgorod, Russia); Sergey V. Rodimov, Victor B. Filimonov, Andrey A. Melnikov, Igor A. Suchkov (Ryazan State Medical University, Ryazan, Russia); EvgeniyS. Drozdov, Dmitriy N. Kostromitskiy (Siberian State Medical University, Tomsk, Russia); Olle Sjöström (Östersund Hospital, Östersund, Sweden); Peter Matthiessen, Bayar Baban, Soran Gadan, Kaveh Dehlaghi Jadid (chool of Medical Sciences, Örebro University, Örebro, Sweden); Maria Staffan (Region Dalarna Hospital, Dalarna University, Falun, Sweden); Jennifer M. Park, Daniel Rydbeck (Scandinavian Surgical Outcomes Research Group, Institute of Clinical Sciences, Sahlgrenska Academy, University of Gothenburg, Gothenburg, Sweden, Region Västra Götaland, Sahlgrenska University Hospital/Östra, Gothenburg, Sweden); Marie-Louise Lydrup, Pamela Buchwald, Henrik Jutesten, Lotten Darlin, Ebba Lindqvist (Skåne Univeristy Hospital, Malmö, Sweden); Karl Nilsson, Per-Anders Larsson (Skaraborgs Hospital, Skövde, Sweden); 186 Staffan Jangmalm (Växjö Hospital, Växjö, Sweden); Jurij A. Košir, Aleš Tomažič, Jan Grosek, Tajda Košir Božič (Ljubljana University Medical Centre, Ljubljana, Slovenia); Aya Zazo, Rama Zazo, Hala Fares, Kusay Ayoub (University of Aleppo, Aleppo, Syria); Ammar Niazi, Ali Mansour, Ayman Abbas, Mohammad Tantoura (The Arabic Medicine Hospital, Aleppo, Syria); Alaa Hamdan, Naya Hassan, Bassam Hasan, Ahmad Saad (Tishreen University, Latakia, Syria); Amine Sebai, Anis Haddad, Houcine Maghrebi, Montasser Kacem (La Rabta Hospital, Tunis, Tunisia); Ömer Yalkın, Mehmet Veysi Samsa, İbrahim Atak (Ali Osman Sönmez Oncology Hospital, Bursa, Turkiye); Bengi Balci, Elifcan Haberal, Lütfi Dogan (Ankara Oncology Training and Research Hospital, Ankara, Turkiye); Ibrahim E. Gecim, Cihangir Akyol, Mehmet A. Koc (Ankara University Medical School, Ankara, Turkiye); Emre Sivrikoz, Deniz Piyadeoğlu (Bahçeşehir University, Istanbul, Turkiye); John O. Larkin, Dara O. avanagh (St. James’s, Hospital, Dublin, Ireland); Selman Sökmen, Tayfun Bişgin, Erşan Günenç, Melek Güzel (Dokuz Eylul University, Balcova, Izmir, Turkiye); Sezai Leventoğlu, Osman Yüksel, Ramazan Kozan, Hüseyin Göbüt (Gazi University Medical School, Ankara, Turkiye); Fevzi Cengiz, Kemal Erdinc, Nihan Coşgun Acar, Erdinc Kamer (Izmir Katip Celebi University, İzmir, Turkiye); İlker Özgür, Oguzhan Aydın, Metin Keskin, Mehmet Türker Bulut, Cemil B. Kulle (Istanbul University, Istanbul Faculty of Medicine, Istanbul, Turkiye); Yasin Kara, Osman Sıbıç (University of Health Sciences, Kanuni Sultan Suleyman Training and Research Hospital, Istanbul, Turkiye); İbrahim H. Özata, Dursun Buğra, Emre Balık, Cemil B. Kulle (Koç University Hospital, Istanbul, Turkiye); Murat Çakır, Anas Alhardan (Meram Tip Faculty Hospital, Meram/Konya, Turkiye); Elif Colak, Ahmet B. Ciftci, Engin Aybar, Ahmet Can Sari (University of Samsun, Samsun Training and Research Hospital, Samsun, Turkiye); Semra Demirli Atici, Tayfun Kaya, Ayberk Dursun, Bulent Calik (University of Health Sciences, Tepecik Training and Research Hospital, Izmir, Turkiye); Ömer Faruk Özkan, Hanife Şeyda Ülgür, Özgül Düzgün (University of Health Sciences Turkiye, Ümraniye Training and Research Hospital, Istanbul, Turkiye); John Monson, Sarah George, Kayla Woods (AdventHealth Orlando, Orlando, Florida, USA); Fatima Al-Eryani, Rudaina Albakry (Al-Kuwait Hospital, Sana’a, Yemen); Emile Coetzee (Life St. George’s Hospital, Port Elizabeth, Eastern Cape, South Africa); Adam Boutall, Ayesiga Herman, Claire Warden, Naser Mugla (Groote Schuur Hospital and University of Cape Town, Cape Town, South Africa); Tim Forgan, Imraan Mia, Anton Lambrechts (Tygerberg Academic Hospital, Parow, Cape Town, South Africa).

## Supplementary Material

znaf189_Supplementary_Data

## Data Availability

Data may be available upon reasonable request.

## References

[1] Lohsiriwat V, Jitmungngan R. Rectovaginal fistula after low anterior resection: prevention and management. World J Gastrointest Surg 2021;13:764–771 34512900 10.4240/wjgs.v13.i8.764 PMC8394379

[2] Woo IT, Park JS, Choi GS, Park SY, Kim HJ, Lee HJ. Optimal strategies of rectovaginal fistula after rectal cancer surgery. Ann Surg Treat Res 2019;97:142–148 31508395 10.4174/astr.2019.97.3.142 PMC6722289

[3] Watanabe J, Ota M, Kawaguchi D, Shima H, Kaida S, Osada S et al Incidence and risk factors for rectovaginal fistula after low anterior resection for rectal cancer. Int J Colorectal Dis 2015;30:1659–1666 26248793 10.1007/s00384-015-2340-5

[4] Greijdanus NG, Wienholts K, Ubels S, Talboom K, Hannink G, Wolthuis A et al Stoma-free survival after anastomotic leak following rectal cancer resection: worldwide cohort of 2470 patients. Br J Surg 2023;110:1863–1876 37819790 10.1093/bjs/znad311 PMC10638542

[5] Kosugi C, Saito N, Kimata Y, Ono M, Sugito M, Ito M et al Rectovaginal fistulas after rectal cancer surgery: incidence and operative repair by gluteal-fold flap repair. Surgery 2005;137:329–336 15746788 10.1016/j.surg.2004.10.004

[6] Barugola G, Bertocchi E, Leonardi A, Almoudaris AM, Ruffo G. Post surgical rectovaginal fistula: who really benefits from stoma diversion? Updates Surg 2021;73:165–171 32449033 10.1007/s13304-020-00810-w

[7] Fleshner PR, Schoetz DJ, Roberts PL, Murray JJ, Coller JA, Veidenheimer MC. Anastomotic-vaginal fistula after colorectal surgery. Dis Colon Rectum 1992;35:938–943 1395980 10.1007/BF02253495

[8] Lamazza A, Fiori E, Sterpetti AV. Endoscopic placement of self-expandable metal stents for treatment of rectovaginal fistulas after colorectal resection for cancer. Gastrointest Endosc 2014;79:1025–1027 24565070 10.1016/j.gie.2014.01.010

[9] Komori K, Kinoshita T, Oshiro T, Ouchi A, Ito S, Abe T et al Surgical strategy for rectovaginal fistula after colorectal anastomosis at a high-volume cancer center according to image type and colonoscopy findings. Anticancer Res 2019;39:5097–5103 31519621 10.21873/anticanres.13704

[10] Göttgens KW, Smeets RR, Stassen LP, Beets G, Breukink SO. The disappointing quality of published studies on operative techniques for rectovaginal fistulas: a blueprint for a prospective multi-institutional study. Dis Colon Rectum 2014;57:888–898 24901691 10.1097/DCR.0000000000000147

[11] van Workum F, Talboom K, Hannink G, Wolthuis A, de Lacy BF, Lefevre JH et al Treatment of anastomotic leakage after rectal cancer resection: the TENTACLE–Rectum study. Colorectal Dis 2021;23:982–988 33169512 10.1111/codi.15435 PMC8246753

[12] Greijdanus NG, Wienholts K, Ubels S, Talboom K, Hannink G, Wolthuis A et al Stoma-free survival after rectal cancer resection with anastomotic leakage: development and validation of a prediction model in a large international cohort. Ann Surg 2023;278:772–780 37498208 10.1097/SLA.0000000000006043 PMC10549897

[13] Von Elm E, Altman DG, Egger M, Pocock SJ, Gøtzsche PC, Vandenbroucke JP. The Strengthening the Reporting of Observational Studies in Epidemiology (STROBE) statement: guidelines for reporting observational studies. The lancet 2007;370:1453–1457 10.1016/S0140-6736(07)61602-X 18064739

[14] Rahbari NN, Weitz J, Hohenberger W, Heald RJ, Moran B, Ulrich A et al Definition and grading of anastomotic leakage following anterior resection of the rectum: a proposal by the international study group of rectal cancer. Surgery 2010;147:339–351 20004450 10.1016/j.surg.2009.10.012

[15] Wienholts K, Sharabiany S, de Wilt JHW, Hompes R, Tanis PJ. Reactivation leakages following stoma reversal after rectal cancer surgery: an underestimated problem. BJS Open 2024;8:zrad150 38170893 10.1093/bjsopen/zrad150 PMC10763996

[16] Potolicchio A, Jehaes C, Merlot B, Assenat V, Dennis T, Roman H et al Treatment techniques for rectovaginal fistulas after low rectal resection for deep endometriosis. Tech Coloproctol 2024;28:51 38684547 10.1007/s10151-024-02923-5

[17] Maspero M, Lavryk O, Prien C, Bandi B, Holubar SD, Gunter R et al Two-stage Turnbull-Cutait pull-through coloanal anastomosis for recurrent rectovaginal fistula. Dis Colon Rectum 2024;67:e244 38150290 10.1097/DCR.0000000000003128

[18] Araujo SEA, Tustumi F, Portilho AS, Horcel L, Edmond Seid V. Laparoscopic redo endorectal pull-through procedure for complex rectovaginal fistula after rectal resection for endometriosis: a video vignette. Colorectal Dis 2023;25:2284–2285 37840227 10.1111/codi.16772

[19] Ellis CN . Outcomes after repair of rectovaginal fistulas using bioprosthetics. Dis Colon Rectum 2008;51:1084–1088 18478298 10.1007/s10350-008-9339-8

[20] Blok RD, Hagemans JAW, Klaver CEL, Hellinga J, van Etten B, Burger JWA et al A systematic review and meta-analysis on omentoplasty for the management of abdominoperineal defects in patients treated for cancer. Ann Surg 2020;271:654–662 30921047 10.1097/SLA.0000000000003266

[21] Pinto RA, Peterson TV, Shawki S, Davila GW, Wexner SD. Are there predictors of outcome following rectovaginal fistula repair? Dis Colon Rectum 2010;53:1240–1247 20706066 10.1007/DCR.0b013e3181e536cb

[22] Corte H, Maggiori L, Treton X, Lefevre JH, Ferron M, Panis Y. Rectovaginal fistula: what is the optimal strategy? An analysis of 79 patients undergoing 286 procedures. Ann Surg 2015;262:855–861 26583676 10.1097/SLA.0000000000001461

[23] Drefs M, Schömer Cuenca S, Wirth U, Kühn F, Burian M, Werner J et al Predictors of outcome for treatment of enterovaginal fistula: therapeutical strategies for treatment. Int J Colorectal Dis 2023;38:187 37420132 10.1007/s00384-023-04453-2 PMC10329052

[24] Pitel S, Lefèvre JH, Tiret E, Chafai N, Parc Y. Redo coloanal anastomosis: a retrospective study of 66 patients. Ann Surg 2012;256:806–811 23095625 10.1097/SLA.0b013e318272de70

[25] Pastier C, Loriau J, Denost Q, O’Connell LV, Challine A, Collard MK et al Rectovaginal fistula: what is the role of Martius flap and gracilis muscle interposition in the therapeutic strategy? Dis Colon Rectum 2024;67:1056–1064 38653492 10.1097/DCR.0000000000003148

