# Supplementary material for: Colorectal-vaginal fistula after rectal cancer resection: international comparative cohort study of characteristics and treatment
Source: Br J Surg. 2025 Nov 18;112(11):znaf189. doi: 10.1093/bjs/znaf189 (PMC12624856; doi:10.1093/bjs/znaf189)
Supplement: znaf189_Supplementary_Data [file znaf189_supplementary_data.docx]

**Colorectal-vaginal fistula after rectal cancer resection; an international comparative cohort study of characteristics and treatment**

Authors:

Mila L. van Lieshout MD^1*^, Jobbe M.G. Lemmens MD^1*^, Nynke G. Greijdanus MD^1^, Kiedo Wienholts MD^2,3,4^, Sander Ubels MD^1,5^, Kevin Talboom MD^2,3,4^, Gerjon Hannink PhD^6^, Albert Wolthuis MD PhD^7^, F. Borja de Lacy MD PhD^8^, Jérémie H. Lefevre MD PhD^9^, Michael Solomon MSc DMed^10^, Matteo Frasson MD PhD^11^, Nicolas Rotholtz MD PhD^12^, Quentin Denost MD PhD^13^, Rodrigo O. Perez MD PhD^14^, Tsuyoshi Konishi MD PhD^15^, Yves Panis MD PhD^16^, Martin Rutegård MD PhD^17^, Roel Hompes MD PhD^2,3,4^, Frans van Workum MD PhD^5^, Pieter J. Tanis MD PhD^2,3,4,18^, Johannes H.W. de Wilt MD PhD^1^

^*^ Shared first authorship

^1^Department of Surgery, Radboud University Medical Center, Radboud Institute for Health Sciences, Nijmegen, the Netherlands ^2^Department of Surgery, Amsterdam University Medical Center, University of Amsterdam, The Netherlands ^3^Cancer Center Amsterdam, Treatment and Quality of Life, Amsterdam, The Netherlands ^4^Cancer Center Amsterdam, Imaging and Biomarkers, Amsterdam, The Netherlands ^5^Department of Surgery, Canisius Wilhelmina Hospital, Nijmegen, the Netherlands ^6^Department of Medical Imaging, Radboud University Medical Center, Radboud Institute for Health Sciences, Nijmegen, the Netherlands ^7^Department of Surgery, UZ Leuven, Leuven, Belgium ^8^Gastrointestinal Surgery Department, Hospital Clinic of Barcelona, University of Barcelona, Barcelona, Spain ^9^Department of Digestive Surgery, Sorbonne Université, AP-HP, Hôpital Saint Antoine, Paris, France ^10^Department of Surgery, University of Sydney Central Clinical School, Camperdown, New South Wales, Australia ^11^Department of Surgery, Hospital La Fe, University of Valencia, Spain ^12^Department of Surgery, Hospital Alemán, Buenos Aires, Argentina ^13^Bordeaux Colorectal Institute, Clinique Tivoli, Bordeaux, France ^14^Colorectal Surgery, Hospital Alemão Oswaldo Cruz, São Paulo, Brazil ^15^Department of Colon and Rectal Surgery, The University of Texas MD Anderson Cancer Center, Anderson, United States of America ^16^Colorectal Surgery Center, Groupe Hospitalier Privé Ambroise Paré-Hartmann, Neuilly Seine, France ^17^Diagnostics and Intervention, Surgery, Umeå University, Umeå, Sweden ^18^Department of Surgical Oncology and Gastrointestinal Surgery, Erasmus Medical Center, Rotterdam, the Netherlands

Collaborators: Andreas J.A. Bremers, Floris T. Ferenschild (Radboud University Medical Centre, Radboud Institute for Health Sciences, Nijmegen, The Netherlands) Stefanie de Vriendt, André D’Hoore, Gabriele Bislenghi (University Hospitals Leuven, Leuven, Belgium); Jordi Farguell, Antonio M. Lacy, Paula González Atienza (Hospital Clínic de Barcelona, Barcelona, Spain); Charlotte S. van Kessel (Royal Prince Albert Hospital, Sydney, Australia); Yann Parc, Thibault Voron, Maxime K. Collard (Sorbonne Université, AP-HP, Hôpital Saint Antoine, Paris, France); Jorge Sancho Muriel, Hannia Cholewa (Valencia University Hospital La Fe, Valencia, Spain); Laura A. Mattioni (Hospital Alemán, Buenos Aires, Argentina); Alice Frontali (Beaujon Hospital, Clichy, and University of Paris, Clichy, France); Sebastiaan W. Polle, Fatih Polat, Ndidi J. Obihara (Canisius Wilhelmina Hospital, Nijmegen, the Netherlands); Bruna B. Vailati (Hospital Alemão Oswaldo Cruz, São Paulo, Brazil); Miranda Kusters, Jurriaan B. Tuynmann, Sanne J.A. Hazen, Alexander A.J. Grüter (Amsterdam University Medical Centers, location VUmc, Amsterdam, The Netherlands; Cancer Center Amsterdam, Treatment and Quality of Life, Amsterdam, The Netherlands; Cancer Center Amsterdam, Imaging and Biomarkers, Amsterdam, The Netherlands); Takahiro Amano, Hajime Fujiwara (Cancer Institute Hospital of the Japanese Foundation for Cancer Research, Tokyo, Japan); Mario Salomon, Hernán Ruiz, Ricardo Gonzalez, Diego Estefanía (Buenos Aires British Hospital, Buenos Aires, Argentina); Nicolas Avellaneda, Augusto Carrie, Mateo Santillan (CEMIC University Hospital, Buenos Aires, Argentina); Diana A. Pantoja Pachajoa, Matias Parodi, Manuel Gielis (Clínica Universitaria Reina Fabiola, Córdoba, Argentina); Alf-Dorian Binder, Thomas Gürtler, Peter Riedl (Universitätsklinikum Tulln, Tulln an der Donau, Austria); Sarit Badiani, Christophe Berney, Matthew Morgan (Bankstown-Lidcombe Hospital, Sydney, New South Wales, Australia); Paul Hollington, Nigel da Silva, Gavin Nair (Flinders Medical Centre, Adelaide, South Australia, Australia); Yiu M. Ho, Michael Lamparelli, Raj Kapadia (Rockhampton Hospital, Queensland, Australia); 19 Hidde M. Kroon, Nagendra N. Dudi-Venkata, Jianliang Liu, Tarik Sammour (Royal Adelaide Hospital, Adelaide, South Australia, Australia); Nicolas Flamey, Paul Pattyn, Ahmed Chaoui, Louis Vansteenbrugge (AZ Delta, Roeselare, Belgium); Nathalie E.J. van den Broek, Patrick Vanclooster, Charles de Gheldere (Heilig-Hartziekenhuis, Lier, Belgium); Pieter Pletinckx, Barbara Defoort, Maxime Dewulf (Maria Middelares Ghent, Belgium); Mihail Slavchev, Nikolay Belev, Boyko Atanasov, Panche Krastev (University Hospital Eurohospital - Medical University Plovdiv, Plovdiv, Bulgaria); Manol Sokolov, Svilen Maslyankov, Petar Gribnev, Vasil Pavlov (Aleksandrovska University Hospital, Sofia, Bulgaria); Tsvetomir Ivanov, Martin Karamanliev, Emil Filipov, Pencho Tonchev (Medical University Pleven, Pleven, Bulgaria); Felix Aigner, Martin Mitteregger, Caterina Allmer, Gerald Seitinger (St. John of God Hospital Graz, Graz, Austria); Nicola Colucci, Nicolas Buchs, Frédéric Ris, Christian Toso (Geneva University Hospitals and Faculty of Medicine, Geneva, Switzerland); Eleftherios Gialamas, Aurélie Vuagniaux, Roland Chautems, Marc-Olivier Sauvain (Neuchâtel Hospital, Neuchâtel, Switzerlandl); Silvio Daester, Markus von Flüe, Marc-Olivier Guenin, Stephanie Taha-Mehlitz, Gabriel F. Hess (St. Clara Hospital and University Hospital Basel, Basel, Switzerland); Lubomír Martínek, Matej Skrovina, Maria Machackova, Vladimir Benčurik (Hospital Nový Jičín, Nový Jičín, Czech Republic); Deniz Uluk, Johann Pratschke, Luca S. Dittrich, Safak Guel-Klein (Charité-Universitätsmedizin Berlin, Corporate Member of Freie Universität Berlin and Humboldt-Universität zu Berlin and Berlin Institut of Health, Berlin, Germany); Daniel Perez (Asclepios Clinic Altona, Hamburg, Germany); Julia-Kristin Grass, Nathaniel Melling, Simone Mueller (University Medical Centre of Hamburg-Eppendorf, Hamburg, Germany); Lene H. Iversen, Jacob D. Eriksen (Aarhus University Hospital, Aarhus, Denmark); Gunnar Baatrup, Issam Al-Najami, Thomas Bjørsum-Meyer (Odense University Hospital, Svendborg Sygehus, Denmark); Jüri Teras, Roland M. Teras (North Estonia Medical Centre Foundation, Tallinn, Estonia); Fatma A. Monib, Nagm Eldin Abu Elnga Ahmed, Eithar Alkady, Ahmed K. Ali (Assiut University Hospital, Assiut, Egypt); Gehan Abd Elatti Khedr, Ahmed Samir Abdelaal, Fouad M. Bassyouni Ashoush, Moataz Ewedah (Alexandria Main University Hospital, Alexandria Governorate, Egypt); Eslam M. Elshennawy, Mohamed Hussein (Kafr Elshikh University Hospital, Kafr el-Sheikh, Egypt); Daniel Fernández-Martínez, Luis J. García-Flórez, María Fernández-Hevia, Aida Suárez-Sánchez (Central University Hospital of Asturias, Asturias, Spain); Izaskun del Hoyo Aretxabala, Iria Losada Docampo, Jesús Gómez Zabala (Basurto University Hospital, Bilbao, Spain); Patricia Tejedor, Javier T. Morales Bernaldo de Quirós, Ignacio Bodega Quiroga (Hospital Universitario Gómez Ulla, Spain); Antonio Navarro-Sánchez, Iván Soto Darias, Cristina López Fernández, Cristina de La Cruz Cuadrado (Hospital Materno Infantil de Gran Canaria, Las Palmas, Spain); Luis Sánchez-Guillén, Francisco López-Rodríguez-Arias, Álvaro Soler-Silva, Antonio Arroyo (University Hospital of Elche, Elche, Spain); Juan C. Bernal-Sprekelsen, Segundo Á. Gómez-Abril, Paula Gonzálvez, María T. Torres (Hospital Universitario Dr. Peset, Valencia, Spain); Teresa Rubio Sánchez, Francisco Blanco Antona, Juan E. Sánchez Lara, José A. Alcázar Montero (University Hospital of Salamanca, Salamanca, Spain); Fernando Mendoza-Moreno, Manuel Díez-Alonso, Belén Matías-García, Ana Quiroga-Valcárcel (Hospital Universitario Príncipe de Asturias, Spain); Enrique Colás-Ruiz, Marta M. Tasende-Presedo, Ignacio Fernández-Hurtado, José A. Cifuentes-Ródenas, Marta Castro Suárez (Son Llàtzer Hospital, Illes Balears, Spain); Manuel Losada, Miguel Hernández, Alfredo Alonso, Beatriz Diéguez (Hospital Universitario del Sureste, Madrid, Spain); Daniel Serralta, Rita E. Medina Quintana, Jose M. Gil Lopez, Francisca Lima Pinto, Elena Nieto-Moreno (Hospital Infanta Leonor, San Sebastián de los Reyes, Madrid, Spain); Alba Correa Bonito, Carlos Cerdán Santacruz, Elena Bermejo Marcos, Javier García Septiem (University Hospital de La Princesa, Madrid, Spain); Aránzazu Calero-Lillo, Javier Alanez-Saavedra, Salvador Muñoz-Collado,, Manuel López-Lara (Fundación Hospital del Espíritu Santo, Santa Coloma de Gramenet, Barcelona, Spain); María Labalde Martínez, Eduardo Ferrero Herrero, Francisco Javier García Borda, Óscar García Villar (12 de Octubre University Hospital, Madrid, Spain); Jorge Escartín, Juan L. Blas, Rocío Ferrer, Jorge García Egea (Hospital Royo Villanova, Zaragoza, Spain); Antonio Rodríguez-Infante, Germán Mínguez-Ruiz, Guillermo Carreño-Villarreal, Gerardo Pire-Abaitua (Hospital Universitario San Agustín, Avilés, Spain); Jana Dziakova, Carlos Sáez-Cazallas Rodríguez, María J. Pizarro Aranda, José M. Muguerza Huguet (Hospital Universitario Clínico San Carlos, Madrid, Spain); Nerea Borda-Arrizabalaga, José M. Enriquez-Navascués, Garazi Elorza Echaniz, Yolanda Saralegui Ansorena (Donostia University Hospital, Donostia, Spain); Mercedes Estaire-Gómez, Carlos Martínez-Pinedo, Alejandro Barbero-Valenzuela, Pablo Ruíz-García (Hospital General Universitario de Ciudad Real, Ciudad Real, Spain); Miquel Kraft, María J. Gómez-Jurado, Gianluca Pellino, Eloy Espín-Basany (Vall d'Hebron University Hospital, Universitat Autonoma de Barcelona, Barcelona, Spain); Eddy Cotte, Nathalie Panel, Claire-Angéline Goutard (Hospices Civils de Lyon, Lyon Sud University Hospital, Pierre Bénite, France); Nicola deÁngelis, Lelde Lauka (Henri Mondor Hospital, AP-HP, Créteil, France); Shafaque Shaikh, Laura Osborne, George Ramsay (Aberdeen Royal Infirmary, NHS Grampian, Aberdeen, United Kingdom); Vladimir-Ion Nichita, Santosh Bhandari, Panchali Sarmah (Cambridgeshire in Peterborough City Hospital, Peterborough, United Kingdom); Rob M. Bethune, Heather C.M. Pringle, Lisa Massey, George E. Fowler (Royal Devon and Exeter Hospital, Exeter, United Kingdom); Hytham K.S. Hamid, Belinda D. de Simone (East Kent Hospitals University NHS Foundation Trust, Ashford, United Kingdom); James Kynaston, Nicholas Bradley, Roxane M. Stienstra (Forth Valley Royal Hospital, Larbert, Scotland); Shashank Gurjar, Tanmoy Mukherjee, Ashfaq Chandio, Safia Ahmed (Bedfordshire Hospitals NHS Foundation Trust, Luton, United Kingdom); Baljit Singh, Francois Runau, Sanjay Chaudhri, Oliver Siaw (Leicester General Hospital, Leicester, United Kingdom); Janahan Sarveswaran, Victor Miu, Daniel Ashmore, Haitham Darwich (Pinderfields Hospital, Wakefield, United Kingdom); Deepak Singh-Ranger, Nirbhaibir Singh (The Royal Wolverhampton NHS Trust, Wolverhampton, West Midlands, United Kingdom); Mohamed Shaban (Newcastle upon Tyne NHS Foundation Trust, Newcastle upon Tyne, United Kingdom); Fahed Gareb (Queen Elizabeth The Queen Mother Hospital, Margate, United Kingdom); Thalia Petropolou, Adreas Polydorou (Euroclinic Athens, Athens, Greece); Mit Dattani, Asma Afzal (University Hospitals Birmingham NHS Foundation Trust, Birmingham, United Kingdom); Akshay Bavikatte, Boby Sebastian, Nicholas Ward, Amitabh Mishra (West Suffolk Hospital, Suffolk, United Kingdom); Dimitrios Manatakis, Christos Agalianos,Nikolaos Tasis, Maria-Ioanna Antonopoulou (Athens Naval and Veterans Hospital, Athens, Greece); Ioannis Karavokyros, Alexandros Charalabopoulos, Dimitrios Schizas, Efstratia Baili, Athanasios Syllaios, Lysandros Karydakis, Michail Vailas (Laikon General Hospital- National and Kapodistrian University of Athens, Greece); Dimitrios Balalis, Dimitrios Korkolis, Aris Plastiras, Aliki Rompou (Saint Savvas Anti-Cancer Hospital, Athens Greece); Sofia Xenaki, Evangelos Xynos, Emmanuel Chrysos, Maria Venianaki (University Hospital of Heraklion Crete, Greece); Grigorios Christodoulidis, Konstantinos Perivoliotis, George Tzovaras, Ioannis Baloyiannis (University Hospital of Larissa, Larissa Greece); Man-Fung Ho, Simon Siu-man Ng, Tony Wing-chung Mak, Kaori Futaba (Prince of Wales Hospital, The Chinese University of Hong Kong, Shatin, Hong Kong); Goran Šantak, Damir Šimleša, Jurica Ćosić, Goran Zukanović (General County Hospital Požega, Požega, Croatia); Michael E. Kelly, John O. Larkin, Paul H. McCormick, Brian J. Mehigan (The Trinity St. James’s Cancer Institute, Dublin + School of Medicine, Trinity College Dublin, Ireland); Tara M. Connelly, Peter Neary, Jessica Ryan, Peter McCullough (University Hospital Waterford, Waterford, Ireland); Maytham A. Al-Juaifari, Hayder Hammoodi, Ali Hashim Abbood (Al-Sadder Teaching Hospital, Najaf, Iraq); Marcello Calabrò, Andrea Muratore, Antonio La Terra, Francesca Farnesi (Edoardo Agnelli Hospital, Pinerolo, Italy); Carlo V. Feo, Nicolò Fabbri, Antonio Pesce, Marta Fazzin (Azienda Unità Sanitaria Locale di Ferrara, Università di Ferrara, Ferrara, Italy); Francesco Roscio, Federico Clerici (ASST Valle Olona Busto Arsizio Italy, Busto Arsizio VA, Italy); Andrea Lucchi, Laura Vittori, Laura Agostinelli, Maria Cristina Ripoli (AUSL Romagna Ceccarini Hospital, Riccione, Italy); Daniele Sambucci, Andrea Porta (Fatebenefratelli Hospital "Holy Family", Erba, Italy); Giovanni Sinibaldi, Giacomo Crescentini, Antonella larcinese, Emanuele Picone (Fatebbenefratelli Hospital, Isola Tiberina, Rome, Italy); Roberto Persiani, Alberto Biondi, Roberto Pezzuto, Laura Lorenzon, Gianluca Rizzo, Claudio Coco, Luca D’Agostino ("A. Gemelli" University Hospital, Catholic University of Rome, Rome, Italy); Antonino Spinelli, Matteo M. Sacchi, Michele Carvello, Caterina Foppa (Humanitas University, Milan, Italy); Antonino Spinelli, Matteo M. Sacchi, Michele Carvello, Caterina Foppa, Annalisa Maroli (IRCCS Humanitas Research Hospital, Milan, Italy); Gian M. Palini, Gianluca Garulli, Nicola Zanini (Infermi Hospital of Rimini, AUSL Della Romagna, Rimini, Italy); Paolo Delrio, Daniela Rega, Fabio Carbone, Alessia Aversano (Fondazione Giovanni Pascale - IRCCS, Naples, Italy); Giovanni Pirozzolo, Alfonso Recordare, Lucrezia D'Alimonte, Chiara Vignotto (Dell'Angelo Hospital, Venice, Italy); Carlo Corbellini, Gianluca M. Sampietro, Leonardo Lorusso, Carlo A. Manzo (ASST Rhodense, Rho Memorial Hospital, Milano, Italy); Federico Ghignone, Giampaolo Ugolini, Isacco Montroni, Franceso Pasini (Ospedale Santa Maria delle Croci, Ravenna, Italy); Francesco Pasini (Ospedale per gli Infermi, Faenza, Italy); Michele Ballabio, Pietro Bisagni, Francesca T. Armao, Marco Longhi (Maggiore Hospital in Lodi, Lodi, Italy); Omar Ghazouani, Raffaele Galleano (Santa Corona Hospital, Pietra Ligure, Italy); Nicolò Tamini, Massimo Oldani, Luca Nespoli (San Gerardo Hospital, Monza, Italy); Arcangelo Picciariello, Donato F. Altomare, Giovanni Tomasicchio, Giuliano Lantone (University of Bari Aldo Moro, Bari, Italy); Fausto Catena, Mario Giuffrida, Alfredo Annicchiarico, Gennaro Perrone (Parma University Hospital, Parma, Italy); Ugo Grossi, Giulio A. Santoro, Giacomo Zanus ,Alessandro Iacomino, Simone Novello, Nicola Passuello, Martino Zucchella (Regional Hospital Treviso, Treviso, Italy); Lucia Puca, Maurizio deGiuli, Rossella Reddavid (San Luigi University Hospital, Orbassano, Torino, Italy); Stefano Scabini, Alessandra Aprile, Domenico Soriero, Emanuela Fioravanti (AOU San Martino Hospital, Genoa, Italy); Matteo Rottoli, Angela Romano, Marta Tanzanu, Angela Belvedere (IRCCS Azienda Ospedaliero Universitaria di Bologna, Bologna, Italy); Nicolò M. Mariani, Andrea P. Ceretti, Enrico Opocher (ASST Santi Paolo e Carlo, Milan, Italy); Gaetano Gallo, Giuseppe Sammarco (University of Catanzaro, Catanzaro, Italy); Gilda de Paola (University of Milano, Milano, Italy); Salvatore Pucciarelli, Francesco Marchegiani, Gaya Spolverato, Gianluca Buzzi (Azienda Ospedale-Università di Padova, Padova, Italy); Salomone Di Saverio, Paola Meroni, Cristiano Parise, Elisa I. Bottazzoli (University of Insubria, University Hospital of Varese, ASST Sette Laghi, Regione Lombardia, Varese, Italy); Pierfrancesco Lapolla, Gioia Brachini, Bruno Cirillo, Andrea Mingoli ("P. Valdoni", Policlinico Umberto I University Hospital, Sapienza University of Rome, Rome, Italy); Giuseppe Sica, Leandro Siragusa, Vittoria Bellato, Daniele Cerbo (University of Rome "Tor Vergata", Rome, Italy); Carlo A. de Pasqual, Giovanni de Manzoni, Maria A. di Cosmo (University of Verona, Verona, Italy); Bourhan M.H. Alrayes, Mahmoud W. M. Qandeel (Islamic Hospital Amman, Amman, Jordan); Mohammad Bani Hani (King Abdullah University Hospital, Ar-Ramtha, Jordan); Alexander Rabadi, Mohammad S. el Muhtaseb, Basel Abdeen, Fahed Karmi (The University of Jordan, Amman, Jordan); Justas Žilinskas, Tadas Latkauskas, Algimantas Tamelis, Ingrida Pikūnienė, Vygintas Šlenfuktas (Hospital of Lithuanian University of Health Sciences Kaunas Clinics, Kaunas, Lithuania); Tomas Poskus, Marius Kryzauskas, Matas Jakubauskas, Saulius Mikalauskas, Lina Jakubauskiene (Vilnius University, Vilnius, Lithuania); Soha Y. Hassan, Amani Altrabulsi (Benghazi Medical Center, Benghazi, Libya); Eman Abdulwahed, Reem Ghmagh, Abdulqudus Deeknah, Entisar Alshareea (Tripoli Central Hospital, Tripoli, Libya); Muhammed Elhadi, Saleh Abujamra, Ahmed A. Msherghi, Osama W.E. Tababa (Tripoli University Hospital, Tripoli, Libya); Mohammed A. Majbar, Amine Souadka, Amine Benkabbou, Raouf Mohsine, Sabrillah Echiguer (National Institute of Oncology, University Mohammed V in Rabat, Rabat, Morocco); Paulina Moctezuma-Velázquez, Noel Salgado-Nesme, Omar Vergara-Fernández, Juan C. Sainz-Hernández, Francisco E. Alvarez-Bautista (Instituto Nacional de Ciencias Médicas y Nutrición Salvador Zubirán, Mexico City, Mexico); Andee D. Zakaria, Zaidi Zakaria, Michael P.K. Wong, Razif Ismail (Universiti Sains Malaysia, Kubang Kerian, Kelantan, Malaysia); Aini F. Ibrahim, Nik A.N. Abdullah, Rokayah Julaihi (Universiti Malaysia Sarawak, Kota Samarahan, Sarawak); Sameer Bhat, Greg O'Grady, Ian Bissett (University of Auckland, Auckland, New Zealand); Bas Lamme, Gijsbert D. Musters, Anne M. Dinaux (Albert Schweitzer Hospital, Dordrecht, The Netherlands); Brechtje A. Grotenhuis, Ernst J. Steller Arend G.J. Aalbers, Marjolein M. Leeuwenburgh (Netherlands Cancer Institute-Antoni van Leeuwenhoek, Amsterdam, The Netherlands); Harm J.T. Rutten, Jacobus W.A. Burger, Johanne G. Bloemen, Stijn H.J. Ketelaers (Catharina Hospital, Eindhoven, The Netherlands); Usama Waqar, Tabish Chawla, Hareem Rauf, Pallavi Rani (Aga Khan University, Karachi City, Pakistan); Aaldert K. Talsma, Lieke Scheurink, Jasper B. van Praagh (Deventer Hospital, Deventer, The Netherlands); Josefin Segelman, Jonas Nygren, Kajsa Anderin, Marit Tiefenthal (Ersta Hospital, Stockholm, Sweden); Beatriz de Andrés, Juan P. Beltrán de Heredia, Andrea Vázquez, Tania Gómez (University Clinical Hospital of Valladolid, Valladolid, Spain); Parisa Golshani, Rawaz Kader, Abudi Mohamed (Gävle Hospital, Gävle, Sweden); Marinke Westerterp, Andreas Marinelli, Quirine Niemer (Medical Center Haaglanden, Westeinde, Den Haag, Netherlands); Pascal G. Doornebosch, Joël Shapiro, Maarten Vermaas, Eelco J.R. de Graaf (Jsselland Hospital, Capelle Aan Den IJssel, The Netherlands); Hendrik L. van Westreenen, Marije Zwakman, Annette D. van Dalsen (Isala Hospital, Zwolle, The Netherlands); Wouter J. Vles, Joost Nonner, Boudewijn R. Toorenvliet, Paul T.J. Janssen (Ikazia Hospital, Rotterdam, the Netherlands); Emiel G.G. Verdaasdonk, Femke J. Amelung (Jeroen Bosch Hospital, 's-Hertogenbosch, The Netherlands); Koen C.M.J. Peeters Renu R. Bahadoer, Fabian A. Holman (Leiden University Medical Center, Leiden, Netherlands); Jeroen Heemskerk, Noortje Vosbeek, Jeroen W.A. Leijtens, Sophie B.M. Taverne (Laurentius Hospital, Roermond, the Netherlands); Bob H.M. Heijnen, Youssef El-Massoudi, Irene de Groot-van Veen (LangeLand Hospital, Zoetermeer, The Netherlands); Christiaan Hoff, Daniela Jou-Valencia (Medical Centre Leeuwarden, Leeuwarden, the Netherlands); Esther C.J. Consten Thijs A. Burghgraef, Ritch Geitenbeek, Lorenzo G.W.L. Hulshof (Meander Medical Centre, Amersfoort, Netherlands); Gerrit D. Slooter, Muriël Reudink (Máxima Medical Centre, Veldhoven, Netherlands); Nicole D. Bouvy, Aurelia C. L. Wildeboer, Sonja Verstappen, Alexander J. Pennings (Maastricht University Medical Centre, Maastricht, The Netherlands); Berber van den Hengel, Allard G. Wijma, Jael de Haan (Martini Hospital, Groningen, The Netherlands); Lindsey C.F. de Nes, Vera Heesink (Maasziekenhuis Pantein, Boxmeer, The Netherlands); Tom Karsten, Charlotte M. Heidsma, Willem J. Koemans (Onze Lieve Vrouwe Gasthuis, Amsterdam, the Netherlands); Jan-Willem T. Dekker, Charlène J. van der Zijden, Daphne Roos (Reinier de Graaf Gasthuis, Delft, The Netherlands); Ahmet Demirkiran, Sjirk van der Burg (Red Cross Hospital, Beverwijk, The Netherlands); Steven J. Oosterling, Tijs J. Hoogteijling (Spaarne Gasthuis, Haarlem, The Netherlands); Bastiaan Wiering, Diederik P.J. Smeeing (Slingeland Ziekenhuis, Doetinchem, Netherlands); Klaas Havenga, Hamid Lutfi, Esther C.J. Consten (University Medical Centre Groningen, Groningen, The Netherlands); Konstantinos Tsimogiannis, Filip Sköldberg, Joakim Folkesson (Uppsala University, Uppsala, Sweden); Frank den Boer, Ted G. van Schaik , Pieter van Gerven (Zaans Medical Center, Zaandam, the Netherlands); Colin Sietses, Jeroen C. Hol (Gelderse Vallei Hospital Ede, Ede, The Netherlands); Evert-Jan G. Boerma, Davy M.J. Creemers (Zuyderland Medical Center, Sittard/Heerlen, The Netherlands); Johannes K. Schultz, Tone Frivold, Rolf Riis (Akershus University Hospital, Lørenskog, Norway); Hilde Gregussen, Sondre Busund (Hospital innland Hamar, Hamar, Norway); Ole H. Sjo, Maria Gaard, Nina Krohn, Amanda L. Ersryd (Ullevål Oslo University Hospital, Oslo, Norway); Edmund Leung (Hereford County Hospital, Hereford, United Kingdom); Usama Waqar, Tabish Chawla, Hareem Rauf, Pallavi Rani (Aga Khan University, Karachi City, Pakistan); Hytham Sultan, Baraa Nabil Hajjaj, Ahmed Jehad Alhisi, Ahmed A.E. Khader (Al-Shifa Hospital, Gaza City, Palestine); Ana Filipa Dias Mendes, Miguel Semião, Luis Queiroz Faria, Constança Azevedo (Centro Hospitalar Universitário Cova da Beira, Covilha, Portugal); Helena M. da Costa Devesa, Sónia Fortuna Martins, Aldo M. Rodrigues Jarimba, Sónia M. Ribeiro Marques (Hospital Distrital de Santarém, Santarém, Portugal); Rita Marques Ferreira, António Oliveira, Cátia Ferreira, Ricardo Pereira (Centro Hospitalar de Trás-os-Montes e Alto Douro EPE, Vila Real, Portugal); Valeriu M. Surlin, Giorgiana M. Graure, Stefan Patrascu Sandu D. Ramboiu (Clinical County Emergency Hospital of Craiova, University of Medicine and Pharmacy of Craiova, Romania); Ionut Negoi, Cezar Ciubotaru, Bogdan Stoica, Ioan Tanase (Carol Davila University of Medicine and Pharmacy Bucharest, Bucharest, Romania); Bogdan Stoica, Cezar Ciubotaru, Valentina M. Negoita (Clinical Emergency Hospital Bucharest, Bucharest, Romania); Sabrina Florea, Florin Macau, Mihai Vasile, Victor Stefanescu (Central Military Emergency Hospital Dr. Carol Davila, Bucharest, Romania); Gabriel-Mihail Dimofte, Sorinel Luncă, Cristian-Ene Roată, Ana-Maria Mușină (Regional Oncology Institute, Iasi, Romania); Tatiana Garmanova, Mikhail N. Agapov, Daniil G. Markaryan, Galliamov Eduard (Lomonosov Moscow State University, Moscow, Russia); Alexey Yanishev, Alexander Abelevich, Andrey Bazaev (Privolzhsky Research Medical University, Nizhny Novgorod, Russia); Sergey V. Rodimov, Victor B. Filimonov, Andrey A. Melnikov, Igor A. Suchkov (Ryazan State Medical University, Ryazan, Russia); EvgeniyS. Drozdov, Dmitriy N. Kostromitskiy (Siberian State Medical University, Tomsk, Russia); Olle Sjöström (Östersund Hospital, Östersund, Sweden); Peter Matthiessen, Bayar Baban, Soran Gadan, Kaveh Dehlaghi Jadid (chool of Medical Sciences, Örebro University, Örebro, Sweden); Maria Staffan (Region Dalarna Hospital, Dalarna University, Falun, Sweden); Jennifer M. Park, Daniel Rydbeck (Scandinavian Surgical Outcomes Research Group, Institute of Clinical Sciences, Sahlgrenska Academy, University of Gothenburg, Gothenburg, Sweden, Region Västra Götaland, Sahlgrenska University Hospital/Östra, Gothenburg, Sweden); Marie-Louise Lydrup, Pamela Buchwald, Henrik Jutesten, Lotten Darlin, Ebba Lindqvist (Skåne Univeristy Hospital, Malmö, Sweden); Karl Nilsson, Per-Anders Larsson (Skaraborgs Hospital, Skövde, Sweden); 186 Staffan Jangmalm (Växjö Hospital, Växjö, Sweden); Jurij A. Košir, Aleš Tomažič, Jan Grosek, Tajda Košir Božič (Ljubljana University Medical Center, Ljubljana, Slovenia); Aya Zazo, Rama Zazo, Hala Fares, Kusay Ayoub (University of Aleppo, Aleppo, Syria); Ammar Niazi, Ali Mansour, Ayman Abbas, Mohammad Tantoura (The Arabic Medicine Hospital, Aleppo, Syria); Alaa Hamdan, Naya Hassan, Bassam Hasan, Ahmad Saad (Tishreen University, Latakia, Syria); Amine Sebai, Anis Haddad, Houcine Maghrebi, Montasser Kacem (La Rabta Hospital, Tunis, Tunisia); Ömer Yalkın, Mehmet Veysi Samsa, İbrahim Atak (Ali Osman Sönmez Oncology Hospital, Bursa, Turkiye); Bengi Balci, Elifcan Haberal, Lütfi Dogan (Ankara Oncology Training and Research Hospital, Ankara, Turkiye); Ibrahim E. Gecim, Cihangir Akyol, Mehmet A. Koc (Ankara University Medical School, Ankara, Turkiye); Emre Sivrikoz, Deniz Piyadeoğlu (Bahçeşehir University, Istanbul, Turkiye); John O. Larkin, Dara O. avanagh (St. James’s, Hospital, Dublin, Ireland); Selman Sökmen, Tayfun Bişgin, Erşan Günenç, Melek Güzel (Dokuz Eylul University, Balcova, Izmir, Turkiye); Sezai Leventoğlu, Osman Yüksel, Ramazan Kozan, Hüseyin Göbüt (Gazi University Medical School, Ankara, Turkiye); Fevzi Cengiz, Kemal Erdinc, Nihan Coşgun Acar, Erdinc Kamer (Izmir Katip Celebi University, İzmir, Turkiye); İlker Özgür, Oguzhan Aydın, Metin Keskin, Mehmet Türker Bulut, Cemil B. Kulle (Istanbul University, Istanbul Faculty of Medicine, Istanbul, Turkiye); Yasin Kara, Osman Sıbıç (University of Health Sciences, Kanuni Sultan Suleyman Training and Research Hospital, Istanbul, Turkiye); İbrahim H. Özata, Dursun Buğra, Emre Balık, Cemil B. Kulle (Koç University Hospital, Istanbul, Turkiye); Murat Çakır, Anas Alhardan (Meram Tip Faculty Hospital, Meram/Konya, Turkiye); Elif Colak, Ahmet B. Ciftci, Engin Aybar, Ahmet Can Sari (University of Samsun, Samsun Training and Research Hospital, Samsun, Turkiye); Semra Demirli Atici, Tayfun Kaya, Ayberk Dursun, Bulent Calik (University of Health Sciences, Tepecik Training and Research Hospital, Izmir, Turkiye); Ömer Faruk Özkan, Hanife Şeyda Ülgür, Özgül Düzgün (University of Health Sciences Turkiye, Ümraniye Training and Research Hospital, Istanbul, Turkiye); John Monson, Sarah George, Kayla Woods (AdventHealth Orlando, Orlando, Florida, United States of America); Fatima Al-Eryani, Rudaina Albakry (Al-Kuwait Hospital, Sana’a, Yemen); Emile Coetzee (Life St. George’s Hospital, Port Elizabeth, Eastern Cape, South Africa); Adam Boutall, Ayesiga Herman, Claire Warden, Naser Mugla (Groote Schuur Hospital and University of Cape Town, Cape Town, South Africa); Tim Forgan, Imraan Mia, Anton Lambrechts (Tygerberg Academic Hospital, Parow, Cape Town, South Africa).

**Corresponding author**: Jobbe Lemmens

*(also for reprints)* Radboud University Medical Center

P.O. Box 9101, 6500 HB, Nijmegen, The Netherlands

Tel: +31 24 361 38 08
 Fax: +31 24 354 05 01

Email: Jobbe.Lemmens@radboudumc.nl
 ORCID ID: 0009-0007-9551-5363, X: @tentaclestudy

**Supplementary Materials – Index**

| **Supplementary Methods** |  |
| --- | --- |
| N/A |  |
| **Supplementary Results** |  |
| N/A |  |
| **Supplementary Appendixes** |  |
|  |  |
| **Supplementary Figures and Tables** |  |
| Table S1: Baseline characteristics of female patients with AL after rectal cancer resection stratified by presence of CRVF. | Pag. 9 |
| Table S2: All treatment and outcome characteristics stratified by presence of CRVF. | Pag. 12 |
| Table S3: Baseline characteristics of female patients with anastomotic leakage and CRVF stratified by 1-year stoma-free survival. | Pag. 14 |
| **References** |  |

N/A

**Table S1 | Baseline characteristics of female patients with AL after rectal cancer resection stratified by presence of CRVF.**

| Variable | CRVF n = 88 (12.7%) | no CRVF  n = 606 (87.3%) | P-value |
| --- | --- | --- | --- |
| **Patient characteristics** |  |  |  |
| **Age (years), median [i.q.r.]** | 65 [56-72] | 63 [54-72] | 0.517 |
| **ASA grade** |  |  | 0.292 |
| I | 16/87 (18.4%) | 113/595 (19.0%) |  |
| II | 54/87 (62.1%) | 322/595 (54.1%) |  |
| III-IV | 17/87 (19.5%) | 160/595 (26.9%) |  |
| **BMI (kg/m^2^)** |  |  | 0.843 |
| Underweight (<18.5) | 8/81 (9.9%) | 56/551 (10.2%) |  |
| Normal (18.5 - 24.9) | 30/81 (37.0%) | 191/551 (34.7%) |  |
| Overweight (25-29.9) | 28/81 (34.6%) | 178/551 (32.3%) |  |
| Obesity (>30) | 15/81 (18.5%) | 126/551 (22.9%) |  |
| **Tumour characteristics** |  |  |  |
| **Clinical tumour stage** |  |  | 0.891 |
| cT0 | 1/86 (1.2%) | 5/577 (0.9%) |  |
| cT1 | 2/86 (2.3%) | 18/577 (3.1%) |  |
| cT2 | 16/86 (18.6%) | 124/577 (21.5%) |  |
| cT3 | 58/86 (67.4%) | 358/577 (62.0%) |  |
| cT4 | 9/86 (10.5%) | 72/577 (12.5%) |  |
| **Clinical metastasis stage** |  |  | 0.768 |
| cM0 | 73/79 (92.4%) | 479/524 (91.4%) |  |
| cM1 | 6/79 (7.6%) | 45/524 (8.6%) |  |
| **Height lower tumour border from ARJ (mm), median [i.q.r.]** | 43 [15-75] | 55 [30-80] | 0.013 |
| **Neoadjuvant therapy** |  |  | 0.194 |
| None | 32/88 (36.4%) | 246/606 (40.6%) |  |
| Radiotherapy only | 6/88 (6.8%) | 80/606 (13.2%) |  |
| Chemotherapy only | 2/88 (2.3%) | 10/606 (1.7%) |  |
| Chemoradiation | 48/88 (54.5%) | 270/606 (44.6%) |  |
| **Surgical characteristics** |  |  |  |
| **Abdominal approach** |  |  | 0.089 |
| Laparoscopic | 45/87 (51.7%) | 362/606 (59.7%) |  |
| Robot-assisted | 4/87 (4.6%) | 48/606 (7.9%) |  |
| Laparotomy | 38/87 (43.7%) | 196/606 (32.3%) |  |
| **Full splenic flexure mobilization** | 54/75 (72.0%) | 316/514 (61.5%) | 0.078 |
| **Multi-visceral resection** | 17/87 (19.5%) | 61/595 (10.3%) | 0.011 |
| Vagina | 11/17 | 6/61 (9.8%) | <0.001 |
| Lateral lymph node(s) | 2/17 | 8/61 (13.1%) | 0.883 |
| Ureter + bladder | 1/17 | 3/61 (4.9%) | 0.873 |
| Uterus | 6/17 | 26/61 (42.6%) | 0.587 |
| Other/unknown | 2/17 | 31/61 (50.8%) | <0.001 |
| **Configuration of anastomosis** |  |  | <0.001 |
| End-end | 42/86 (48.8%) | 376/580 (64.8%) |  |
| Side-end | 35/86 (40.7%) | 178/580 (30.7%) |  |
| Colonpouch | 5/86 (5.8%) | 22/580 (3.8%) |  |
| Coloplasty | 3/86 (3.5%) | 1/580 (0.2%) |  |
| IPAA | 1/86 (1.2%) | 3/580 (0.5%) |  |
| **Technique of anastomosis** |  |  | 0.967 |
| Double stapled | 55/84 (65.5%) | 395/588 (67.2%) |  |
| Double purse string single stapled | 19/84 (22.6%) | 119/588 (20.2%) |  |
| Hand-sewn | 11/84 (13.1%) | 77/588 (13.1%) |  |
| **Anastomosis from ARJ (cm), median [i.q.r.]** | 2 [1-4] | 3 [1-4] | 0.463 |
| **Primary diverting stoma** | 64/88 (72.7%) | 358/606 (59.1%) | 0.014 |
| **Leakage characteristics** |  |  |  |
| **qSOFA** |  |  | 0.041 |
| 0 | 78/84 (92.9%) | 457/567 (80.6%) |  |
| 1 | 4/84 (4.8%) | 82/567 (14.5%) |  |
| 2 | 1/84 (1.2%) | 23/567 (4.1%) |  |
| 3 | 1/84 (1.2%) | 5/567 (0.9%) |  |
| **POD of AL diagnosis, median [i.q.r.]** | 18 [8-55] | 7 [4-15] | <0.001 |
| **Location of the leak** |  |  | 0.032 |
| Circular | 57/59 (96.6%) | 336/393 (85.5%) |  |
| Blind loop | 3/59 (5.1%) | 61/393 (15.5%) |  |
| **Anastomotic defect circumference** |  |  | 0.311 |
| 0-25% | 23/43 | 127/260 (48.8%) | 0.573 |
| 25-50% | 9/43 | 81/260 (31.2%) | <0.001 |
| 50-75% | 8 /43 | 28/260 (10.8%) | 0.141 |
| 75-100% | 3/43 | 24/260 (9.2%) | 0.631 |
| **Leakage location** |  |  | <0.001 |
| Anterior | 22/30 | 41/165 (24.8%) |  |
| Lateral | 4/30 | 44/165 (26.7%) |  |
| Posterior | 4/30 | 80/165 (48.5%) |  |
| **Ischemia of the efferent colon** | 8/75 (10.7%) | 57/490 (11.6%) | 0.807 |
| **Abdominal contamination** | 17/85 (20.0%) | 233/548 (42.5%) | <0.001 |
| **Reactivation leakage** | 18/56 (32.1%) | 42/423 (9.9%) | <0.001 |
| **Hospital characteristics** |  |  |  |
| **Annual procedure volume of hospital** |  |  | 0.439 |
| Low (<20) | 6/88 (6.8%) | 61/606 (10.1%) |  |
| Middle (20-49) | 27/88 (30.7%) | 155/606 (25.6%) |  |
| High (>50) | 55/88 (62.5%) | 390/606 (64.4%) |  |

Values are presented as number with percentage unless otherwise indicated. Numbers and percentages may not add up to 100% due to missing data and to multiple options possible per patient. CRVF, Colorectal-vaginal fistula; AL, Anastomotic leakage; I.Q.R., Interquartile range; ASA, American Society of Anaesthesiology; BMI, Body mass index; ARJ, Anorectal Junction; MVR, Multi-visceral resection; IPAA, Ileal pouch anal anastomosis; qSOFA, quick sequential organ failure assessment; POD, Post-operative days.

**Table S2 | All treatment and outcome characteristics stratified by presence of CRVF.**

| Variable | CRVF n = 88 (12.7%) | no CRVF n = 606 (87.3%) | P-value |
| --- | --- | --- | --- |
| **Treatment modalities** |  |  |  |
| Conservative management of AL (incl. antibiotics and transanal manual drainage on the ward) | 21/88 (23.9%) | 191/606 (31.5%) | 0.215 |
| Radiological reintervention of AL at any time | 3/88 (3.4%) | 73/606 (12.0%) | 0.015 |
| Endoscopic reintervention of AL at any time | 9/88 (10.2%) | 65/606 (10.7%) | 0.887 |
| Surgical reintervention of AL at any time | 65/88 (73.9%) | 329/606 (54.3%) | <0.001 |
| **Total number of reinterventions (median [range])*** | 148/88 (1[0-6]) | 1127/606 (1 [0-10]) |  |
| Total number of non-surgical reinterventions (endoscopic or radiological) (mean/SD) | 12/88 (0 [0-2]) | 202/606 (0 [0-6]) |  |
| Total number of surgical reinterventions (mean/SD) | 136/88 (1[0-6]) | 925/606 (1 [0-10]) |  |
| **Type of surgical reinterventions (total, % patient)** |  |  |  |
| Dismantling of the anastomosis | 24/65 (36.9%) |  |  |
| APR | 7/65 (10.8%) |  |  |
| Re-do of the anastomosis | 9/65 (13.8%) |  |  |
| Abdominal lavage | 7/65 (10.8%) |  |  |
| Transanal drainage | 12/65 (18.5%) |  |  |
| Transanal closure | 6/65 (9.2%) |  |  |
| Diverting stoma | 21/65 (32.3%) |  |  |
| Reversal of diverting stoma | 37/65 (56.9%) |  |  |
| Revision | 6/65 (9.2%) |  |  |
| Other | 7/65 (10.8%) |  |  |
| **POD of first surgical intervention, median [i.q.r.]** | 22 [10-166] | 7 [4-16] | <0.001 |
| **1-year stoma-free survival** | 26/88 (29.5%) | 260/534 (48.7%) | 0.002 |
| **Total duration of hospital stay within 1 year (days), median [i.q.r.]** | 10 [7-20] | 15 [8-25] | 0.013 |
| **Patients admitted to ICU within 1 year** | 29/87 (33.3%) | 227/600 (37.8%) | 0.417 |
| **Duration of ICU stay within 1 year (days), median [i.q.r.]** | 3 [1-6] | 3 [2-6] | 0.453 |
| **Time to leak healing within 1 year (days), median [i.q.r.]** | 160 [83-276] | 145 [69-231] | 0.428 |

Values are presented as number with percentage unless otherwise indicated. Numbers and percentages may not add up to 100% due to missing data and to multiple options possible per patient. *Note: Interventions include treatment of fistula, including all stoma related interventions. The CRVF patients were evaluated individually in treatment, resulting in more accurate numbers of treatment. CRVF, Colorectal-vaginal fistula; I.Q.R., Interquartile range; ICU, Intensive care unit; POD, Postoperative day.

**Table S3 | Baseline characteristics of female patients with anastomotic leakage and CRVF stratified by 1-year stoma-free survival.**

| Variable | Stoma-free after 1 year n = 26 (29.5%) | Stoma after 1 year n = 62 (70.5%) | P-value |
| --- | --- | --- | --- |
| **Patient characteristics** |  |  |  |
| **Age (years), median [i.q.r.]** | 63 [57-72] | 66 [55-72] | 0.777 |
| **ASA grade** |  |  | 0.430 |
| I | 4/25 | 12/62 (19.4%) |  |
| II | 18/25 | 36/62 (58.1%) |  |
| III-IV | 3/25 | 14/62 (22.6%) |  |
| **BMI (kg/m^2^)** |  |  | 0.513 |
| Underweight (<18.5) | 1/26 | 7/55 (12.7%) |  |
| Normal (18.5 – 24.9) | 10/26 | 20/55 (36.4%) |  |
| Overweight (25-29.9) | 11/26 | 17/55 (30.9%) |  |
| Obesity (>30) | 4/26 | 11/55 (20.0%) |  |
| **Tumour characteristics** |  |  |  |
| **Height lower tumour border from ARJ (mm), median [i.q.r.]** | 39 [15-52] | 50 [16-80] | 0.471 |
| **Neoadjuvant therapy** |  |  | 0.924 |
| None | 9/26 | 23/62 (37.1%) |  |
| Radiotherapy only | 2/26 | 4/62 (6.5%) |  |
| Chemotherapy only | 1/26 | 1/62 (1.6%) |  |
| Chemoradiation | 14/26 | 34/62 (54.8%) |  |
| **Operation characteristics** |  |  |  |
| **Abdominal approach** |  |  | 0.409 |
| Laparoscopic | 14/26 | 31/61 (50.8%) |  |
| Robot-assisted | 0 | 4/61 (6.6%) |  |
| Laparotomy | 12/26 | 26/61 (42.6%) |  |
| **Full splenic flexure mobilization** | 20/24 | 34/51 (66.7%) | 0.134 |
| **Multi-visceral resection** | 7/26 | 10/61 (16.4%) | 0.257 |
| Vagina | 6/7 | 5/10 | 0.129 |
| Lateral lymph node(s) | 0 | 2/10 | 0 |
| Ureter + bladder | 1/7 | 0 | 0 |
| Uterus | 2/7 | 4/10 | 0.627 |
| Other/unknown | 0 | 2/10 | 0 |
| **Configuration of anastomosis** |  |  | 0.683 |
| End-end | 12/26 | 30/60 (50%) |  |
| Side-end | 12/26 | 23/60 (38.3%) |  |
| Colonpouch | 2/26 | 3/60 (5.0%) |  |
| Colonplasty | 0 | 3/60 (5.0%) |  |
| IPAA | 0 | 1/60 (1.7%) |  |
| **Technique of anastomosis** |  |  | 0.333 |
| Double stapled | 19/26 | 36/58 (62.1%) |  |
| Double purse string single-stapled | 3/26 | 15/58 (25.9%) |  |
| Hand-sewn | 4/26 | 7/58 (12.1%) |  |
| **Anastomosis from ARJ (cm), median [i.q.r.]** | 1.5 [1-2] | 3 [1-4] | 0.058 |
| **Primary diverting stoma** | 19/26 | 45/62 (72.6%) | 0.962 |
| **Leakage characteristics** |  |  |  |
| **POD of AL diagnosis, median [i.q.r]** | 19 [12-37] | 15 [7-61] | 0.487 |
| **Location of the leak** |  |  | 0.966 |
| Circular | 18/19 | 2/40 |  |
| Blind loop | 1/19 | 38/40 |  |
| **Anastomotic defect circumference** |  |  | 0.030 |
| 0-25% | 12/15 | 11/28 | 0.011 |
| 25-50% | 3/15 | 6/28 | 0.913 |
| 50-75% | 0 | 8/28 | 0 |
| 75-100% | 0 | 3/28 | 0 |
| **Leakage location** |  |  | 0.357 |
| Anterior | 10/14 | 12/16 |  |
| Lateral | 3/14 | 1/16 |  |
| Posterior | 1/14 | 3/16 |  |
| **Abdominal contamination** | 3/26 | 14/59 (23.7%) | 0.195 |
| **Reactivation leakage** | 4/24 | 14/32 | 0.032 |
| **Hospital characteristics** |  |  |  |
| **Annual procedure volume of hospital** |  |  | 0.715 |
| Low (<20) | 1/26 | 5/62 (8.1%) |  |
| Middle (20-49) | 9/26 | 18/62 (29.0%) |  |
| High (>50) | 16/26 | 39/62 (62.9%) |  |

Values are presented as number with percentage unless otherwise indicated. Numbers and percentages may not add up to 100% due to missing data and to multiple options possible per patient. CRVF, Colorectal-vaginal fistula; AL, Anastomotic leakage; I.Q.R., Interquartile range; ASA, American Society of Anaesthesiology; BMI, Body mass index; ARJ, Anorectal Junction; MVR, Multi-visceral resection; IPAA, Ileal pouch anal anastomosis; qSOFA, quick sequential organ failure assessment; POD, Post-operative days.
